# Halotolerant biofilm-producing rhizobacteria mitigate seawater-induced salt stress and promote growth of tomato

**DOI:** 10.1038/s41598-022-09519-9

**Published:** 2022-04-04

**Authors:** Md. Manjurul Haque, Md. Sanaullah Biswas, Md Khaled Mosharaf, Md. Amdadul Haque, Md. Shahidul Islam, Kamrun Nahar, Md. Mynul Islam, Habibul Bari Shozib, Md. Mariful Islam, Ferdous-E-Elahi

**Affiliations:** 1 Department of Environmental Science, Faculty of Agriculture, Bangabandhu Sheikh Mujibur Rahman Agricultural University, Gazipur, 1706 Bangladesh; 2 Department of Horticulture, Faculty of Agriculture, Bangabandhu Sheikh Mujibur Rahman Agricultural University, Gazipur, 1706 Bangladesh; 3 Science Research Center, Yamaguchi University, Yoshida 1677-1, Yamaguchi, 753-8515 Japan; 4 Department of Agro-Processing, Faculty of Agriculture, Bangabandhu Sheikh Mujibur Rahman Agricultural University, Gazipur, 1706 Bangladesh; 5 Bangladesh Jute Research Institute, Manik Mia Avenue, Dhaka, 1207 Bangladesh; 6 Biotechnology Division, Bangladesh Agricultural Research Institute, Joydebpur, Gazipur, 1701 Bangladesh; 7 Plant Pathology Division, Bangladesh Agricultural Research Institute, Joydebpur, Gazipur, 1701 Bangladesh; 8 Grain Quality and Nutrition Division, Bangladesh Rice Research Institute, Joydebpur, Gazipur, 1701 Bangladesh

**Keywords:** Microbiology, Physiology, Plant sciences

## Abstract

Biofilm-producing rhizobacteria (BPR) enhance productivity and mitigate abiotic stresses in plants. This study showed that 21 out of 65 halotolerant rhizobacteria could build biofilms. The components of the biofilm matrices i.e., extracellular polymeric substances (EPS) are proteins, curli, nanocelloluse, nucleic acids, lipids, and peptidoglycans. Various functional groups including carbonyl, carboxyl, amino, hydroxyl, and phosphate were identified. Positions of these groups were shifted by application of 5% NaCl, suggesting Na^+^ biosorption. By sequencing, *Glutamicibacter arilaitensis* (ESK1, ESM4 and ESM7), *G. nicotianae* (ESK19, ESM8 and ESM16), *Enterobacter ludwigii* (ESK15, ESK17, ESM2 and ESM17), *E. cloacae* (ESM5 and ESM12), *Exiguobacterium acetylicum* (ESM24 and ESM25), *Staphylococcus saprophyticus* ESK6, *Leclercia adecarboxylata* ESK12, *Pseudomonas poae* ESK16, *Bacillus subtilis* ESM14, and *P. putida* ESM17 were identified. These rhizobacteria exhibited numerous plant growth-promoting (PGP) activities including producing IAA, ACC deaminase, and siderophores, and solubilizing phosphate. Under non-stress, bacterized plants increased biomass accumulation (8–23.2% roots and 23–49.4% shoots), while under seawater-induced salt stress only ESK12, ESM4, ESM12, and ESM14 enhanced biomass production (5.8–52.9% roots and 8.8–33.4% shoots). Bacterized plants induced antioxidant defense system (19.5–142% catalase and 12.3–24.2% DPPH radical scavenging activity), retained a greater relative water content (17–124%), showed lesser membrane injuries (19.9–26.5%), and a reduced Na^+^ (6–24% in roots) and increased K^+^/Na^+^ ratio (78.8 and 103% in roots by ESK12 and ESM24, respectively) than the non-bacterized plants in saline conditions. Thus, native halotolerant BPR can be utilized as ameliorators of salt stress.

## Introduction

Salinity stress is one of the foremost abiotic stressors affecting agricultural productivity and food security worldwide. Currently, the salinity affected agricultural land worldwide covers over 62 million (20%) hectares^1^, and it will probably be increased about 50% by 2050^2^. In Bangladesh, about 63% of the cultivable land in the coastal regions is affected by different levels of soil salinity^3^. However, Dasgupta et al.^4^ predicted that the annual median, wet- and dry-season soil salinity in Bangladesh will likely be increased to 39.2, 36.6 and 13.1%, respectively by 2050 compared with the baseline year of 2009. Another prediction is that the sea-level rise will likely continue throughout the twenty-first century, which will increase salinity in the coastal regions of countries like Bangladesh^5^. Thus, developing effective strategies for an efficient management of soil salinity is urgently needed for sustainable crop production.

Salinity stress creates osmotic stress that impairs seed germination, plant growth, yield, photosynthesis, gas exchange, water and nutrient uptake, as well as the ionic and hormonal balance in plants^6–9^. Furthermore, salinity stress controls nitrogen metabolism and amino acid production by regulating the expression of different cellular enzymes including RNase, DNase and proteases^10^. Salinity stress induces oxidative stress by generating reactive oxygen species (ROS) like, superoxide radicals, hydroxyl radicals, hydrogen peroxide and singlet oxygen that damages various biomolecules in plants such as proteins, nucleic acids and lipids^7–9,11–13^. All these effects of salinity stress affect gene expression in plants^14,15^. In order to avoid the harmful effects of salinity stress, plants adopt various mechanisms, such as exclusion and compartmentalization of Na^+^ and Cl^−^ ions into vacuoles or old tissues that reduces the ion toxicity^16,17^, and induction of compatible solutes (e.g., proline, free amino acids and sugars) to minimizes the osmotic stress^18,19^. Plants have both antioxidant enzymes and non-enzymatic antioxidants (like phenolics), polyphenolic compounds (e.g., hydroxycinnamic acids, flavanols, flavonols, flavones and flavanones), ascorbic acids, pigments (e.g., tocopherols, carotenoids, betalains) and anthocyanins that have strong radical scavenging capacity^8,9,20,21^. Nevertheless, for stress homeostasis, stressed plants developed mechanisms to boost the expression of the majority of non-enzymatic and enzymatic antioxidants and to detoxify ROS^8,9,18,20–23^.

In agriculture, various physicochemical methods (e.g., flushing, leaching, scraping, shifting of crop calendar, cultivation of halophytes, and application of gypsum, lime and biochar) are being practiced worldwide to ameliorate the salinity stress. Besides physicochemical methods, salt-tolerant crop varieties could be used. However, traditional breeding is time-consuming. Another future alternative is salt-tolerant transgenic crop varieties. But the development of transgenic crop varieties through gene transfer is associated with ethical and social acceptance issues. To counteract these problems, simple and accessible technology should be developed for low-income countries like Bangladesh. One such alternative technology is plant growth-promoting rhizobacteria (PGPR), particularly native halotolerant PGPR to increase plant resilience against salinity stress.

Free-living advantageous bacteria that inhabit the soil around plant roots and stimulate growth are known as PGPR. Numerous PGPR isolated from cereals, pulses and vegetables were found salinity tolerant (halotolerant), and promote plant growth^2,14,24,25^. Halotolerant PGPR form biofilms, and produce extracellular polymeric substances (EPS), compatible solutes, and halophilic enzymes to survive under saline conditions^26,27^. They express various plant growth-promoting (PGP) attributes, such as diazotrophic nitrogen (N) fixation, nutrient solubilization [e.g., phosphate (P), iron (Fe), zinc (Zn) and potassium (K)], indole-3-acetic acid (IAA) production, production of 1-aminocyclopropane-1-carboxylate (ACC) deaminase [ACCD, (this enzyme lowers the ethylene level by metabolizing its precursor ACC into α-ketobutyrate and ammonia)], ammonia (NH_3_), hydrogen cyanide (HCN), EPS, and a biofilm to promote plant growth^1,28–31^. Therefore, the halotolerant rhizobacteria with diverse PGP characteristics, such as the production of IAA, ACC, osmoprotectants, antioxidants, volatile compounds, and especially the formation of biofilm enclosed with EPS are potentially important to alleviate salt stress in plants.

Biofilms are structured surface-associated microbial cells, enclosed with the self-produced matrix of hydrated EPS. On a dry basis, most biofilms contained over 90% of EPS and less than 10% of microbial cells^27^. The biofilm matrix/EPS protects microorganisms against hostile environmental stressors including, drought, salinity, heat, pH, biocides, antibiotics, metallic cations, and ultraviolet radiation^27,32^. The bacterial biofilm matrix consists of a heterogeneous mixture of proteins, polysaccharides, nucleic acids, and lipids^32,33^. These components were reported to act in initial colonization (abiotic and biotic surfaces), aggregation, to maintain a highly hydrated microenvironment, resistance to non-specific and specific host defenses, detoxification of xenobiotics, as a source of carbon (C), N and P, for horizontal gene transfer, to store excess C, and for accumulation, retention and stabilization of enzymes^32,34^. The EPS contains various ligands/chemical functional groups including carboxyl, hydroxyl and amino owing to the presence of proteins, polysaccharides and lipids^35–38^. Some studies have claimed that the functional groups bind Na^+^ thereby reducing the salt stress in plants^39–42^. Bacterial biofilm formation in the roots of crop plants was demonstrated to enhance the production of PGP substances, nutrient cycling, and biocontrol phytopathogens, leading to an increased crop productivity and yield^2,26,43^.

Several researchers have claimed that numerous PGPR that perform well in the laboratory and/or greenhouse conditions are often failed under field conditions^43,44^. On the other hand, Timmusk et al.^45^ reported that native biofilm-producing PGPR are resistant to different abiotic and biotic stressors and performed better under field conditions. It was also shown that biofilm-producing PGPR are much more effective for the expression of PGP traits (e.g., diazotrophic N fixation, IAA synthesis, P and Fe solubilization, and ammonia production), production of antimicrobial compounds, and resistance to antibiotics than the planktonic or non-biofilm-forming PGPR^46–48^. Thus, native biofilm-producing PGPR would be the best choice as inoculants when facing an intense competition with indigenous soil microorganisms and/or harsh environmental conditions. However, only a few biofilm-producing PGPR were identified that promoted the growth and yield of rice, wheat, maize, cucumber, legumes and tomato under favorable conditions^26,43^. Efforts have also been made to isolate salt-tolerant EPS-producing PGPR (e.g., *Aeromonas hydrophila/caviae, Bacillus aryabhattai*, *B. tenquilensis*, *B. licheniformis*, *B. coagulans*, *B. insolitus*, *Burkholderia cepacia*, *Enterobacter* sp., *Halomonas* sp., *Microbacterium* sp., *Paenibacillus macerans, Pseudomonas aeruginosa* and *Rhizobacterium* sp.) without considering the biofilm formation from rice, wheat, maize, pigeon pea, sunflower, canola, quinoa, mint, and chickpea that promoted the plant growth under salt stress^39,40,42,49^.

Tomato (*Solanum lycopersicum* L.) is an important horticultural crop grown worldwide including in Bangladesh as it is rich in minerals and antioxidants^50,51^. The threshold salinity of the most vegetable crops is ≤ 2.5 dS m^−1^. Tomato is highly sensitive to the salinity stress with threshold salinity (dS m^−1^) at 2.5^52^. Therefore, the objectives of this study were to (1) screen and identify halotolerant biofilm/EPS-producing bacteria from the tomato rhizosphere, (2) identify the components and the chemical functional groups in the biofilm matrices, (3) examine the various PGP traits in vitro, and (4) compare the growth of tomato plants under seawater-induced salt stress or non-stress conditions with or without inoculation of halotolerant biofilm-producing PGPR isolates. This study contributes towards an understanding of how biofilm/EPS-producing PGPR isolates may help to promote growth, and ameliorate seawater-induced salt stress in tomatoes.

## Results

### Screening of halotolerant biofilm-producing rhizobacteria

**Figure 1:**
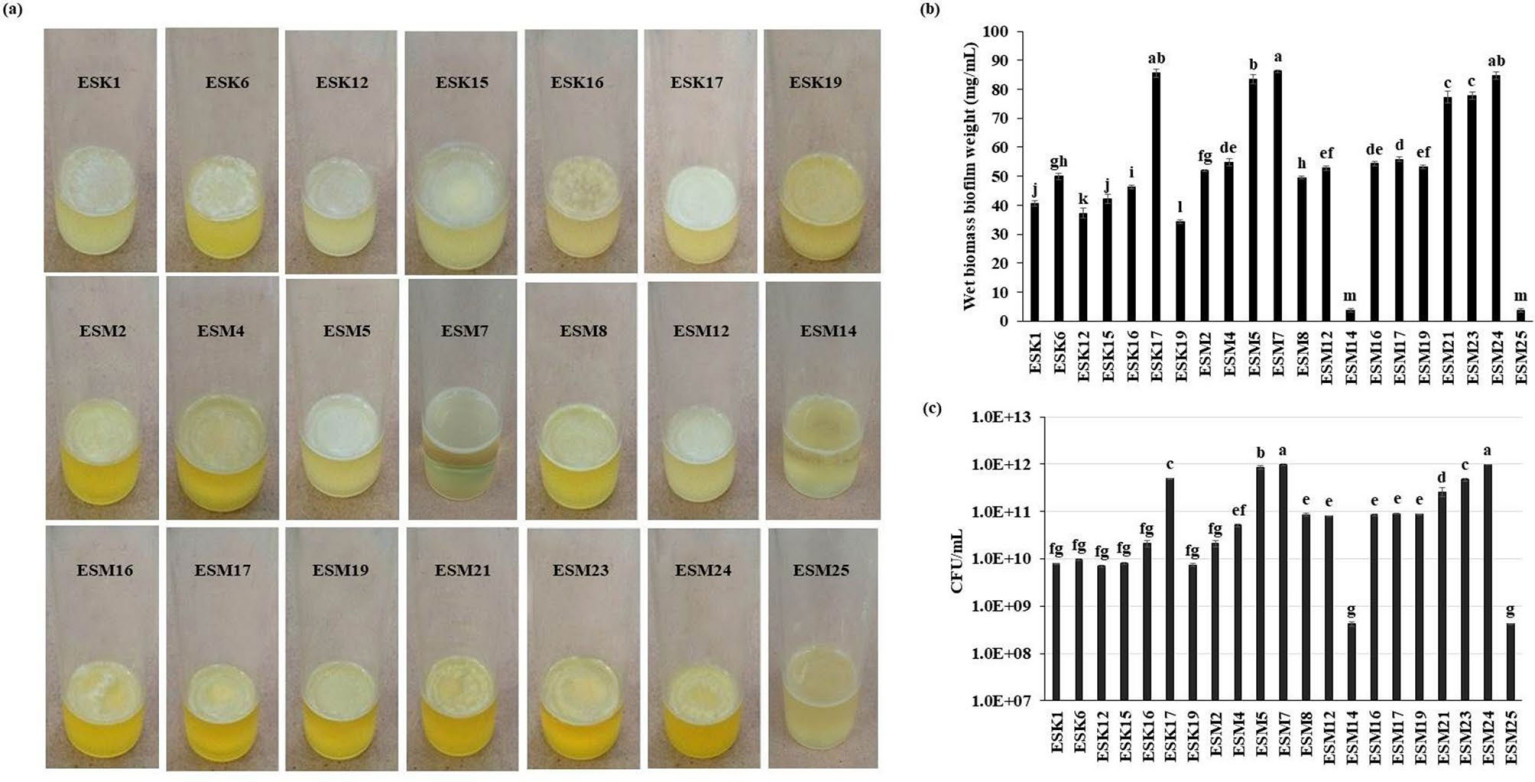
Biofilm formation by different rhizobacterial isolates in the glass test tubes containing 5 mL SOBG supplemented with 5% NaCl after 72 h incubation in a static condition at 28 °C (**a**). Biomass (wet) biofilm production by various isolates (**b**). Number of bacteria associated with biomass biofilm (**c**). The values are mean and error bars indicate standard deviation ( ±) of the three independent experiments. Values having different letters are significantly different from each other according to Fisher’s least significant difference (LSD) test (*P* ≤ 0.001).

A total of 65 halotolerant rhizobacterial isolates were isolated from 13 rhizosphere samples (5 isolates sample^−1^). Biofilm formation by rhizobacteria is one of the important features for ameliorating salinity stress. Thus, all (65) these halotolerant isolates were evaluated for their capacities to form air–liquid (AL)- or solid-air–liquid (SAL) biofilms on the flat bottom glass test tube containing salt-optimized broth plus glycerol (SOBG) media supplemented with 5% NaCl. Among them, 20 isolates (30.7%) built the AL biofilms, while only one isolate (ESM14) formed the SAL biofilm after 72 h incubation in a static condition at 28 °C (Fig. 1A). The remaining isolates neither generated AL nor SAL biofilms even after 2 weeks of incubation (data not shown). Biomass biofilm weight in terms of wet basis was quantified (Fig. 1B). The wet biomass biofilm weight was significantly (*P* ≤ 0.001) varied in these bacterial isolates, and ranged from 3.65 to 86.2 mg mL^−1^. The highest biomass biofilm (86.2 mg mL^−1^) was noted in ESM7 which was statistically similar to ESK17 (85.55 mg mL^−1^) and ESM24 (84.84 mg mL^−1^). Incredibly, the lowest biomass biofilm was obtained in ESM14 (3.65 mg mL^−1^) which was not significantly differed from ESM25 (3.75 mg mL^−1^). Like the production of biomass biofilm, the CFU (colony forming unit mL^−1^) also differed significantly (*P* ≤ 0.001) in the different biofilm matrices, varying from 4.2E+08 to 9.85E+11 (Fig. 1C). The highest CFU was recorded in ESM24 (9.85E+11) though not significantly different from ESM7 (9.6E+11). The second highest CFU was noted in ESM5 (8.5E+11) followed by ESK17 (4.9E+11) and ESM23 (4.7E+11). The lowest CFU (4.2E+08) was found in ESM14 and ESM25.

### Identification of halotolerant biofilm-producing rhizobacteria

**Figure 2:**
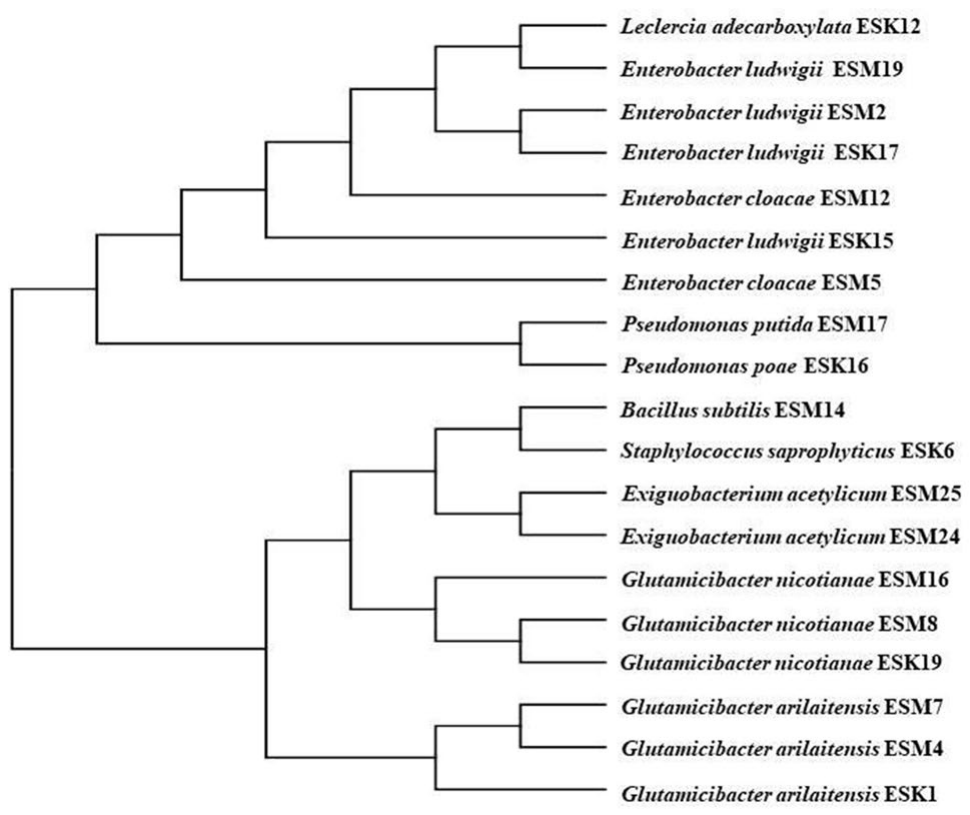
Phylogenetic tree. MUSCLE alignment and maximum likelihood method were performed to construct the tree with Gblock used for alignment refinement.

Because of their negative results in the hemolytic tests, 19 out of 21 biofilm-producing rhizobacteria were sequenced. The isolates ESK1, ESM4 and ESM7 were identified as *Glutamicibacter arilaitensis*; ESK19, ESM8 and ESM16 as *G. nicotianae*; ESK15, ESK17, ESM2 and ESM17 as *Enterobacter ludwigii*; ESM5 and ESM12 as *E. cloacae*; and ESM24 and ESM25 belonged to *Exiguobacterium acetylicum*. The isolates ESK6, ESK12, ESK16, ESM14, and ESM17 were recognized as *Staphylococcus saprophyticus*, *Leclercia adecarboxylata*, *Pseudomonas poae*, *Bacillus subtilis* and *P. putida*, respectively. The 16S rRNA gene sequence data were deposited in the NCBI (National Center for Biotechnology Information) GenBank, and the allotted accession numbers are listed in Table 1. A phylogenetic tree was also constructed using the 16S rRNA gene sequences as presented in Fig. 2.

**Table 1:** Identification of halotolerant biofilm producing rhizobacteria isolated from coastal regions of Bangladesh.

Isolates	Geographical position	Sequence length (bp)	Maximum score	Identity (%)	Top hit against colony isolate	Accession number	Allotted GenBank accession number
**Patuakhali**							
ESK1	21.9814 N; 90.2498 E	1410	2582	99.79	*Glutamicibacter arilaitensis*	MG788347.1	MN173447.1
ESK6	21.9898 N; 90.3219 E	1436	2652	100	*Staphylococcus saprophyticus*	KT260360.1	MN173448.1
ESK12	21.6543 N; 90.2975 E	1420	2606	99.86	*Leclercia adecarboxylata*	CP013990.1	MN173449.1
ESK15	21.9582 N; 90.2792 E	1425	2606	99.79	*Enterobacter ludwigii*	MK830099.1	MN173450.1
ESK16	21.8642 N; 90.2843 E	1404	2593	100	*Pseudomonas poae*	MH211267.1	MN173451.1
ESK17	21.8632 N; 90.2823 E	1406	2597	100	*E. ludwigii*	CP039741.1	MN173452.1
ESK19	21.9395 N; 90.2429 E	1407	2593	99.93	*G. nicotianae*	KY849352.2	MN173453.1
**Bagerhat**							
ESM2	22.4233 N; 89.5877 E	1418	2603	99.86	*E. ludwigii*	CP039741.1	MN173454.1
ESM4	22.4233 N; 89.5877 E	1397	2567	99.86	*G. arilaitensis*	MG788347.1	MN173455.1
ESM5	22.4521 N; 89.5934 E	1418	2599	99.79	*E. cloacae*	MK780068.1	MN173456.1
ESM7	22.4521 N; 89.5934 E	1407	2575	99.72	*G. arilaitensis*	MG788347.1	MN173457.1
ESM8	22.4812 N; 89.6012 E	1408	2571	99.64	*G. nicotianae*	CP033081.1	MN173458.1
ESM12	22.4812 N; 89.6012 E	1421	2606	99.79	*E. cloacae*	KX431213.1	MN173459.1
ESM14	22.4912 N; 89.6098 E	1431	2627	99.86	*Bacillus subtilis*	KX427024.1	MN173460.1
ESM16	22.4912 N; 89.6098 E	1414	2593	99.72	*G. nicotianae*	KX698104.1	MN173461.1
ESM17	22.4812 N; 89.6110 E	1416	2586	99.65	*P. putida*	KJ958211.1	MN173462.1
ESM19	22.4812 N; 89.6110 E	1414	2595	99.79	*E. ludwigii*	MN177186.1	MN173463.1
ESM24	22.4912 N; 89.6018 E	1434	2612	99.72	*Exiguobacterium acetylicum*	KJ146070.1	MN173464.1
ESM25	22.4912 N; 89.6018 E	1439	2652	100	*E. acetylicum*	KJ146070.1	MN173465.1

### Detection of biofilm matrix components and their functional groups

**Figure 3:**
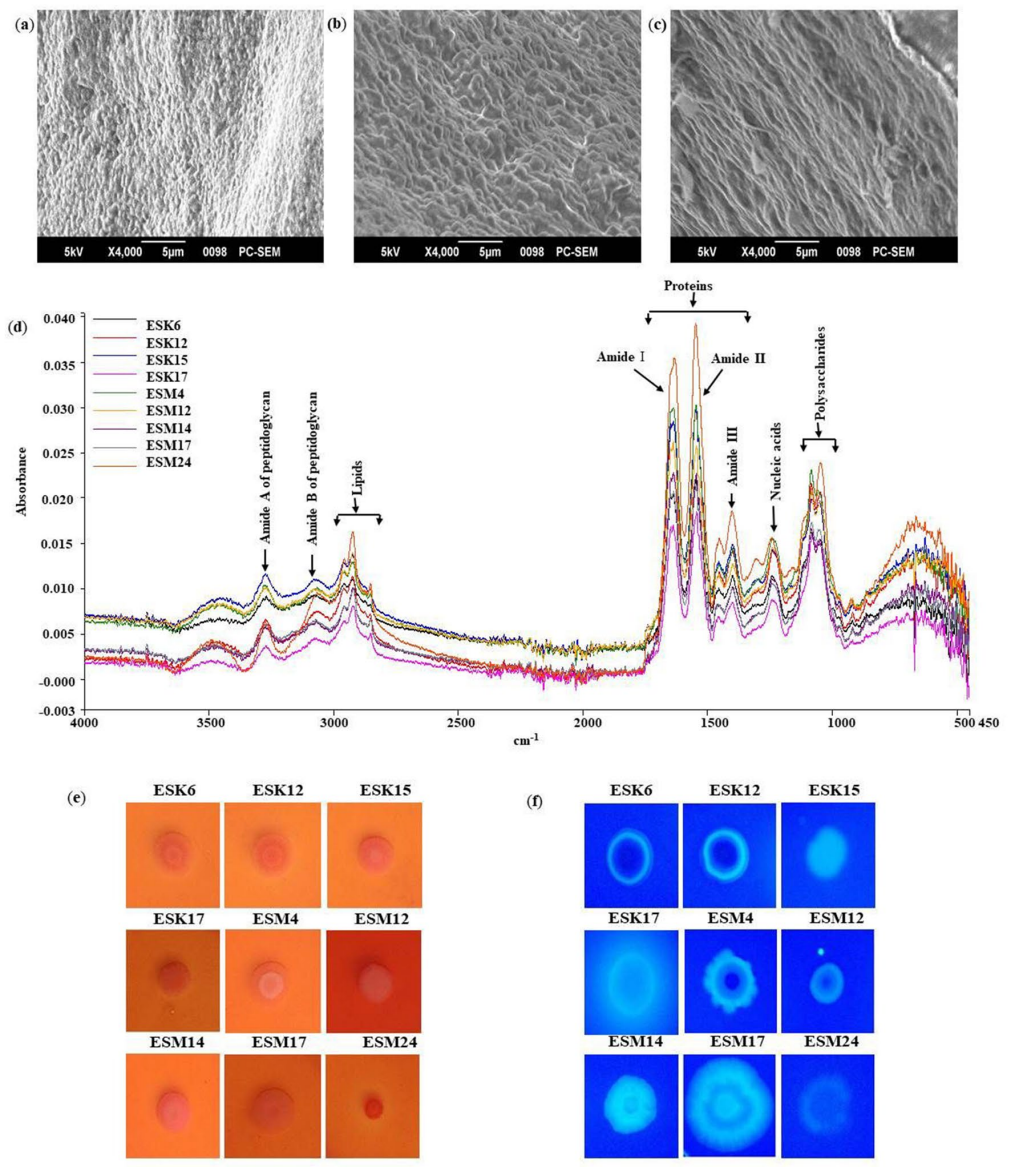
Characterization of the biofilm matrices. Analysis of biofilm matrices from *L. adecarboxylata* ESK12 (**a**), *E. cloacae* ESM5 (**b**) and *E. acetylicum* ESM24 (**c**) by scanning electron microscope. Identification of the biofilm matrix components using FTIR (**d**). Congo red (**e**) and Calcofluor (**f**) binding assays. Photographs (**e**,**f**) taken after 48 h represent one of two experiments, which gave similar results.

The biofilm matrix/EPS components of the identified rhizobacteria were not characterized yet. SEM (scanning electron microscope) was used to characterize the morphology of the biofilm matrices of the selected bacterial isolates. Numerous ribbon-like structures, popularly known as nanocellulose fibers^53^ were observed in the biofilm matrices of *S. saprophyticus* ESK6, *L. adecarboxylata* ESK12, *G. arilaitensis* ESM4, *E. cloacae* ESM5, *P. putida* ESM17 and *E. acetylicum* ESM24. These fibers were highly interconnected and compact. Representative images are illustrated in Fig. 3a–c.

FTIR (Fourier transform infrared) spectroscopy was used to identify the biomolecules and chemical functional groups/ligands present in the biofilm matrices. All the examined biofilm matrices consisted of proteins, polysaccharides, nucleic acids, lipids, and peptidoglycans (Fig. 3d). The peaks of amide I (1600–1700 cm^−1^) and amide II (1500–1600 cm^−1^) were remarkably higher than the peaks of polysaccharides (900–1150 cm^−1^), nucleic acids (1220–1250 cm^−1^), and lipids (2800–2970 cm^−1^). Several functional groups were identified based on available literatures^36–38^. The broad band around 3400 cm^−1^ was related to the O–H stretching mode of the hydroxyl group, while absorptions at 2800–2970 cm^−1^ were assigned to the asymmetrical C–H stretching vibration of the aliphatic CH_2_-group. The bands at 1520–1650 cm^−1^ were attributed to the stretching vibration of C=O, N–H and C–N groups in the presence of proteins, while the band around 1400 cm^−1^ was assigned to C=O symmetric stretching of COO^−^. The bands at 1220–1250 cm^−1^ were characteristics for P=O stretching of PO_2_^−^ phosphodiesters. The peaks at 900–1150 cm^−1^ were correlated to the C–O or C–C stretching vibrations, P=O stretching, and C–O–H or C–O–C deformation vibrations.

Congo red- and Calcofluor (a cellulose-specific dye) binding assays were used to determine whether biofilm producing bacterial isolates produce curli and/or cellulose or not. In this study, all the tested bacterial isolates formed the typical RDAR (red, dry, and rough) phenotype on plates containing Congo red (Fig. 3e) associated with assembly of cellulose and curli^54^. When these isolates were speckled onto the Calcofluor agar plates, they displayed a weak to strong fluorescence (Fig. 3f), suggesting these isolates produced cellulose^55^. However, cellulose production might vary between these bacterial isolates.

### Expression of PGP traits in vitro

**Figure 4:**
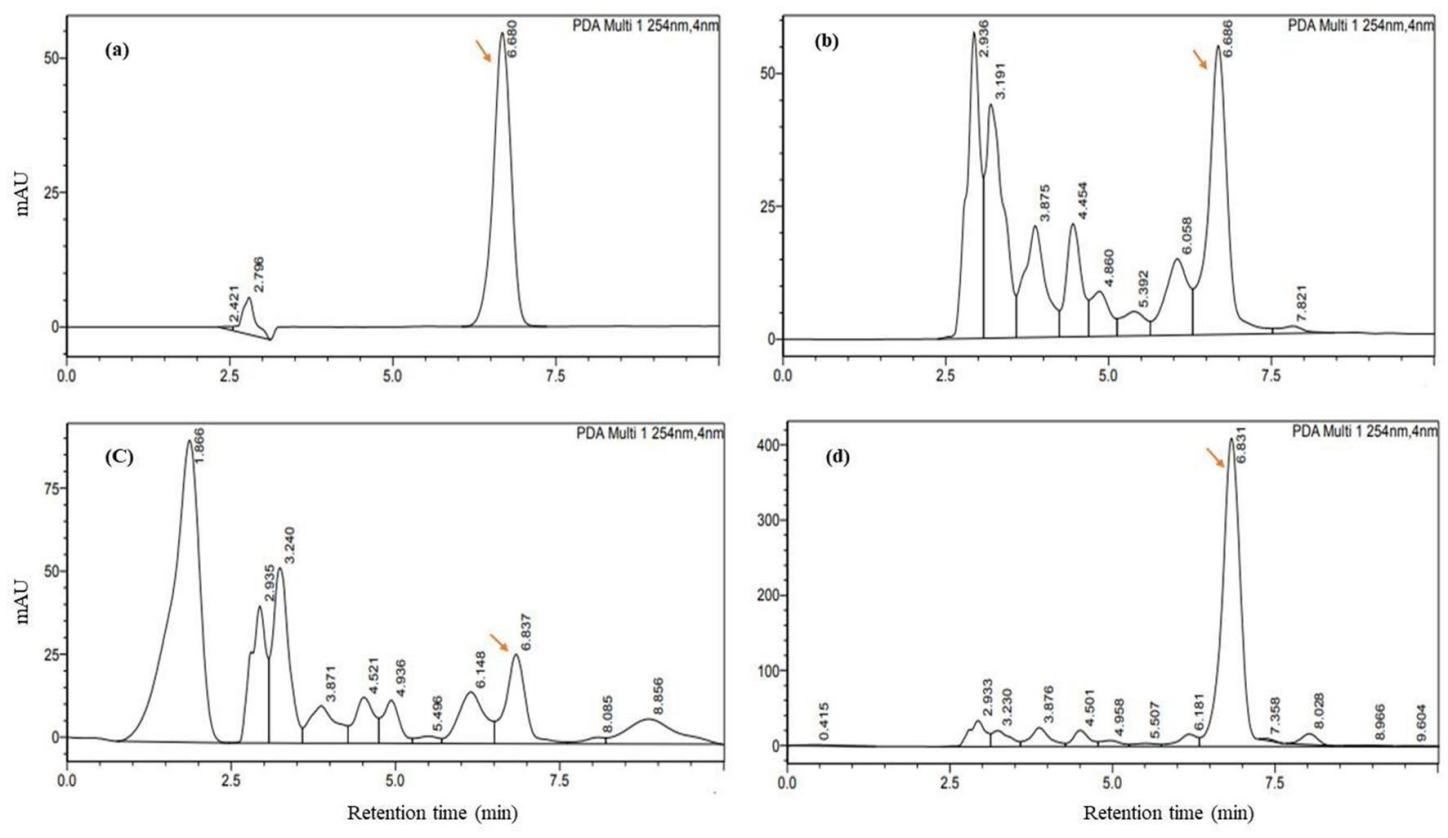
Quantification of IAA by HPLC. Chromatograms of standard (50 µg mL^−1^) IAA (**a**), and IAA in ESM4 (**b**), ESM8 (**c**), and ESM19 (**d**).

IAA is one of the most studied phytohormones secreted by numerous rhizobacteria that stimulate plant growth. IAA production in the presence of 0.2% L-tryptophan was quantified using both Salkowsky colorimetric- and HPLC (High-Performance Liquid Chromatography) methods (Table 2). Except for ESK1 and ESK16, all other cultures developed the pink color after adding the Salkowsky reagent, suggesting a positive auxin production (data not shown). By spectrophotometry, IAA was not detected from nether in ESK1 nor in ESM16. IAA production ranged from 1.56 to 117.47 µg mL^−1 ^in the colorimetric method. In this case, the maximum IAA was produced by ESM19 (117.47 µg mL^−1^) which was statistically similar with ESM2. All the isolates were found to synthesize IAA when quantified using HPLC. A higher amount of IAA was detected in all the isolates by HPLC than by the colorimetric method. IAA production ranged from 19.60 to 384.00 µg mL^−1^ in the HPLC method. In this case, ESM19 synthesized the highest amount of IAA (384 µg mL^−1^) followed by ESM5 (364.01 µg mL^−1^) and ESM2 (337.19 µg mL^−1^). The representative chromatographs of known concentration of IAA, ESM4, ESM8 and ESM19 are shown in Fig. 4a–d.

**Table 2:** Indole-3-acetic acid (IAA) production and nutrient acquisition by different halotolerant biofilm-producing rhizobacterial isolates.

Isolates	IAA production (µg mL^−1^) by		Nitrogen fixation		Phosphate solubilization index		Potassium solubilization	Zinc solubilization
	Spectrophotometric determination	HPLC system	Colony formation on N-free medium	Detection of *nifH*	Tricalcium phosphate	Rock phosphate		
ESK1	−	22.38 ± 1.39 jk	+	+	2.10 ± 0.03 e	1.84 ± 0.14 d	−	−
ESK6	4.04 ± 0.04 l	36.47 ± 1.79 hijk	+	+	2.50 ± 0.24 de	2.83 ± 0.24 c	−	−
ESK12	85.06 ± 1.43 d	125.53 ± 2.94 f.	+	+	4.75 ± 1.77 cd	2.53 ± 0.05 cd	−	−
ESK15	87.11 ± 1.86 d	137.07 ± 6.38 f.	+	+	7.50 ± 0.71 a	1.97 ± 0.05 d	+	−
ESK16	23.85 ± 1.03 h	71.08 ± 4.51 g	+	+	4.08 ± 0.59 cde	2.06 ± 0.08 cd	−	−
ESK17	56.37 ± 1.03 g	127.76 ± 6.79 f.	−	−	3.50 ± 0.24 cde	2.33 ± 0.18 cd	+	−
ESK19	1.93 ± 0.27 lm	19.60 ± 4.46 k	−	−	−	−	−	−
ESM2	117.05 ± 1.71 a	337.19 ± 6.55 c	+	+	2.07 ± 0.0 e	2.15 ± 0.03 cd	−	−
ESM4	11.24 ± 0.25 j	50.34 ± 3.62 h	+	+	2.38 ± 0.18 e	6.73 ± 0.74 a	−	−
ESM5	112.09 ± 1.01 b	364.01 ± 13.38 b	+	+	5.04 ± 0.41 bc	2.65 ± 0.14 cd	−	−
ESM7	1.56 ± 0.24 m	26.31 ± 4.44 jk	+	+	2.67 ± 0.47 de	2.28 ± 0.07 cd	−	−
ESM8	3.13 ± 0.37 lm	28.03 ± 6.07 ijk	+	+	−	−	−	−
ESM12	105.10 ± 1.42 c	261.06 ± 17.50 d	+	+	3.92 ± 0.59 cde	7.20 ± 1.13 a	+	−
ESM14	20.40 ± 1.46 i	80.86 ± 9.32 g	+	+	2.67 ± 0.47 de	2.10 ± 0.14 cd	−	−
ESM16	−	20.17 ± 3.72 k	+	+	−	2.18 ± 0.03 cd	−	−
ESM17	59.06 ± 1.03 f.	122.12 ± 14.61 f.	+	+	2.77 ± 0.04 cde	2.33 ± 0.47 cd	−	−
ESM19	117.47 ± 1.59 a	384.00 ± 19.17 a	+	+	7.25 ± 1.06 a	2.17 ± 0.0 cd	+	−
ESM24	80.56 ± 1.38 e	197.29 ± 6.83 e	+	+	3.50 ± 0.24 cde	5.88 ± 0.18 b	+	+
ESM25	8.20 ± 1.01 k	50.26 ± 9.16 h	+	+	−	−	−	−
ESR15	18.25 ± 1.40	38.78 ± 5.20	+	+	4.46 ± 0.12	4.67 ± 0.0	+	+

The values are mean of three independent experiments. ± indicates standard deviation. Values having different letters are significantly different from each other according to Fisher’s least significant difference (LSD) test (*p* ≤ 0.001). *Pseudomonas chlororaphis* ESR15 was used as positive control.

*IAA* indole-3-acetic acid, *HPLC* high-performance liquid chromatography, + = positive for the respective test, − = negative for the respective test.

In order to prove the isolates were diazotrophs, the *nifH* gene was amplified by polymerase chain reaction (PCR) using *nifH*-specific primers. Except for ESK17 and ESK19, all the isolates amplified 470 bp and 359 bp *nifH* fragments (Supplementary Fig. S1). To confirm the PCR results, the isolates were grown on N-free media. ESK17 and ESK19 did not form any colonies on N-free solid LG agar plates, in contrast to the other isolates. Thus, the majority of the isolates are diazotrophs.

P solubilizing rhizobacteria screened using tricalcium phosphate was found ineffective to solubilize P under field conditions^56^. Hence, P solubilization in terms of P solubilization index (PSI) was compared using both tricalcium phosphate (0.5%) and rock phosphate (0.8%) as a P source (Table 2). Except for ESK19, ESM8 and ESM25, all other isolates were able to solubilize P from both sources. However, ESM16 was incapable to solubilize P from tricalcium phosphate but can solubilize P from rock phosphate. The PSI value varied remarkably (*P* ≤ 0.001) in the different isolates, and fluctuating between 2.07–7.25 and 1.84–7.20 in tricalcium phosphate, and rock phosphate, respectively. Five isolates (ESK15, ESK17, ESM12, ESM19 and ESM24) were capable to solubilize K from potassium aluminum silicate, and only ESM24 solubilized Zn from Zn_3_(PO_4_)_2_ but not from ZnO or ZnCO_3_ (Table 2).

**Figure 5:**
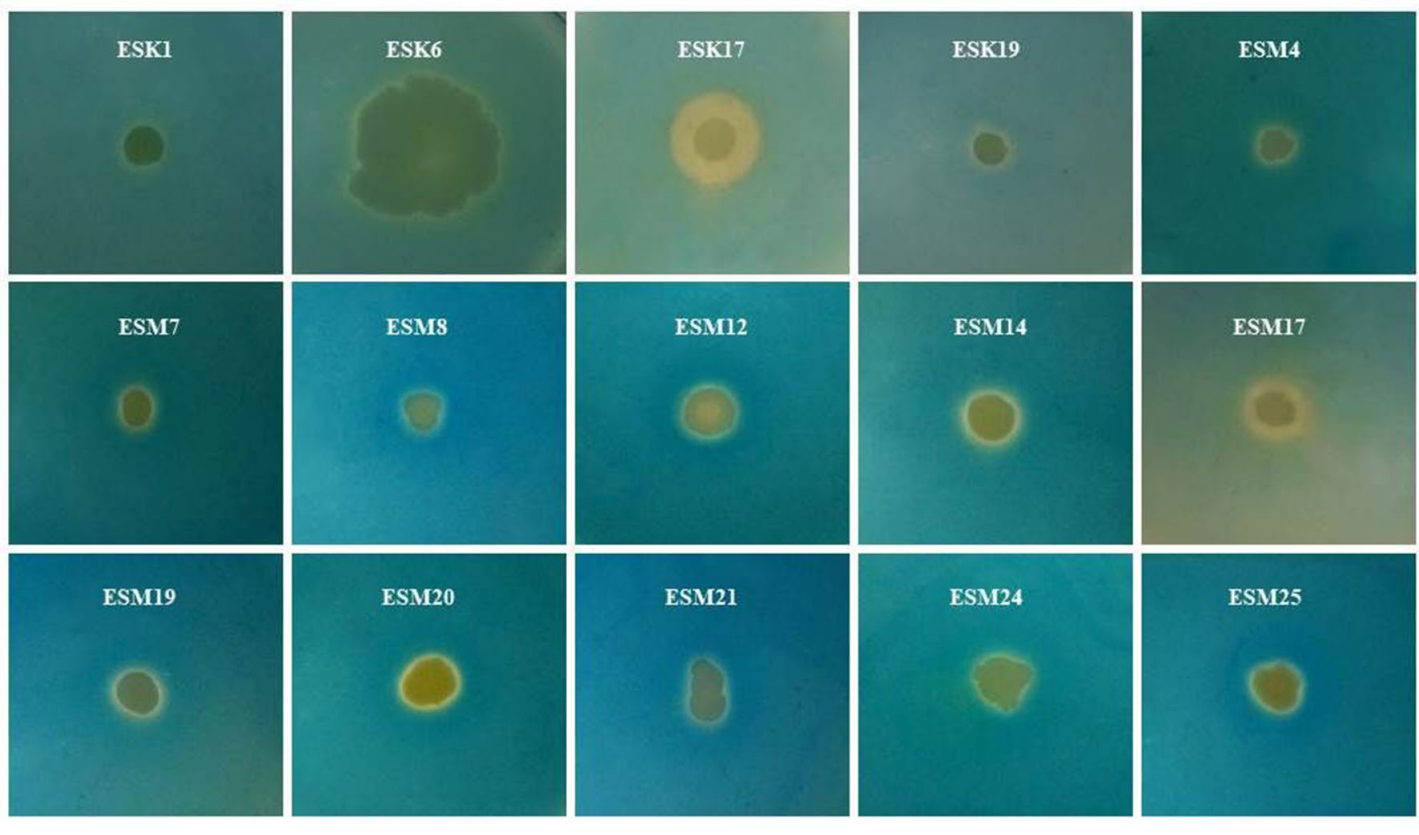
Siderophore production assays. Each isolate (2 µL, ca. 10^7^ CFU mL^−1^) was spotted onto the center of LB agar plates and incubated at 28 °C for 24 h. After applying 8 mL O-CAS broth, those LB agar plates were incubated at 28 °C for another 10 h. Photographs represent one of two experiments, which gave similar results.

Siderophores not only stimulate plant growth, but also act to biocontrol phytopathogens. In fact, three types of siderophores are produced by rhizobacterial strains on the Overlay-Chrome Azurol S (O-CAS) agar medium: catechol (purple), hydroxamate (light orange/orange), carboxylate (light yellow), and both hydroxamate and carboxylate (yellow)^57^. In this study, 15 out of the 19 isolates produced various types of siderophores as presented in Fig. 5. None of the isolates were able to develop the catechol-type siderophore. Isolates ESK17, ESM17, and ESM20 generated the hydroxamate-type siderophore. ESK1, ESK6, ESM8, ESM21, and ESM24 synthesized the carboxylate-type siderophore, and both hydroxamate- and carboxylate-type siderophores were produced by ESK19, ESM4, ESM7, ESM12, ESM14, ESM19, and ESM25.

A vast array of volatile bacterial compounds can improve stress tolerance and/or stimulate plant growth. Here, different volatile compounds including acetoin (also known 3-hydroxybutanone or acetyl methyl carbinol), indole, NH_3_, and HCN produced by different isolates were assayed qualitatively (Table 3 and Supplementary Figs. S2 and S3). Here, 14, 7, 19 and 3 out of the 19 isolates produced acetoin, indole, NH_3,_ and HCN, respectively.

**Table 3:** Qualitative production of volatile compounds and hydrolytic enzymes by various halotolerant biofilm-producing rhizobacterial isolates.

Isolates	Volatile compounds				Hydrolytic enzymes							
	Acetoin	Indole	NH_3_	HCN	ACC deaminase	Catalase	Oxidase	Gelatinase	Dehydrolase	Lipase	Cellulase	Protease
ESK1	−	+	+	−	+	+	−	+	−	−	+	+
ESK6	+	−	+	−	+	+	−	−	−	−	+	−
ESK12	+	+	+	−	+	+	−	+	−	−	+	−
ESK15	+	+	+	−	+	+	+	+	+	−	+	+
ESK16	+	+	+	+	+	+	+	−	+	−	+	−
ESK17	+	−	+	+	+	+	+	−	+	−	+	−
ESK19	+	+	+	−	+	+	−	+	−	+	+	+
ESM2	+	−	+	−	+	+	+	−	+	−	+	−
ESM4	−	−	+	−	+	+	−	−	+	+	+	−
ESM5	+	−	+	−	+	+	+	−	−	−	+	−
ESM7	+	−	+	−	+	+	−	+	+	−	+	+
ESM8	+	−	+	−	+	+	−	+	+	+	+	+
ESM12	+	−	+	−	+	+	+	−	+	−	+	−
ESM14	+	−	+	−	+	+	−	+	+	+	+	+
ESM16	−	+	+	−	+	+	−	+	+	+	+	+
ESM17	−	−	+	+	+	+	+	−	+	−	+	−
ESM19	+	−	+	−	+	+	−	−	+	+	+	−
ESM24	−	−	+	−	+	+	−	−	−	−	+	−
ESM25	+	+	+	−	+	+	+	+	−	−	+	+
ESR15	−	−	+	_+_	+	+	+	+	+	+	+	−

+  = Positive for the respective test, −  = Negative for the respective test. *Pseudomonas chlororaphis* ESR15 was used as control.

Hydrolytic enzymes produced by rhizobacteria act in stress tolerance and in biocontrol of phytopathogens consequently improving plant growth. Table 3 shows the analysis of hydrolytic enzymes. All analyzed isolates synthesized catalases, cellulases and ACCD. Nine isolates (ESK1, ESK12, ESK15, ESK19, ESM7, ESM8, ESM14, ESM16, and ESM25) synthesized gelatinases, while six (ESK19, ESM4, ESM8, ESM14, ESM16, and ESM19) and eight (ESK1, ESK15, ESK19, ESM7, ESM8, ESM14, ESM16, and ESM25) isolates produced lipases and proteases, respectively.

### Abiotic stress tolerance and antagonistic activities

The results of the abiotic stress tolerance analysis are illustrated in Supplementary Table S1. All the isolates grew at 10% NaCl except ESK17, whereas 14 out of the 19 isolates propagated at 15% NaCl on Luria Bertani (LB) agar plates. All the isolates also grew at pH 9 and pH 10. Except for ESK6, ESK12, ESM2, and ESM7, all other isolates grew at the acidic condition of pH 4. Testing of heat tolerance, except for ESK17, all other isolates grew at 42 °C. Interestingly, 14 out of the 19 isolates were able to even grow at 50 °C.

Antagonistic activities of these isolates were also evaluated against *Xanthomonas campestris* pv. *campestris* ATCC 33913, *Ralstonia solanacearum* ATCC® 11696™, and *Pectobacterium carotovorum* subsp. *carotovorum* PCC8 (data not shown). Only ESK17 and ESM17 were able to inhibit the growth of *X. campestris* pv. *campestris* ATCC 33913 (Supplementary Fig. S4), while none of the isolates could stop the progress of *P. carotovorum* subsp. *carotovorum* PCC8 and *R. solanacearum* ATCC® 11696™.

### Biofilm-producing PGPR reduce seawater-induced biochemical changes in tomato plants

#### Electrolyte leakage and relative water content

**Figure 6:**
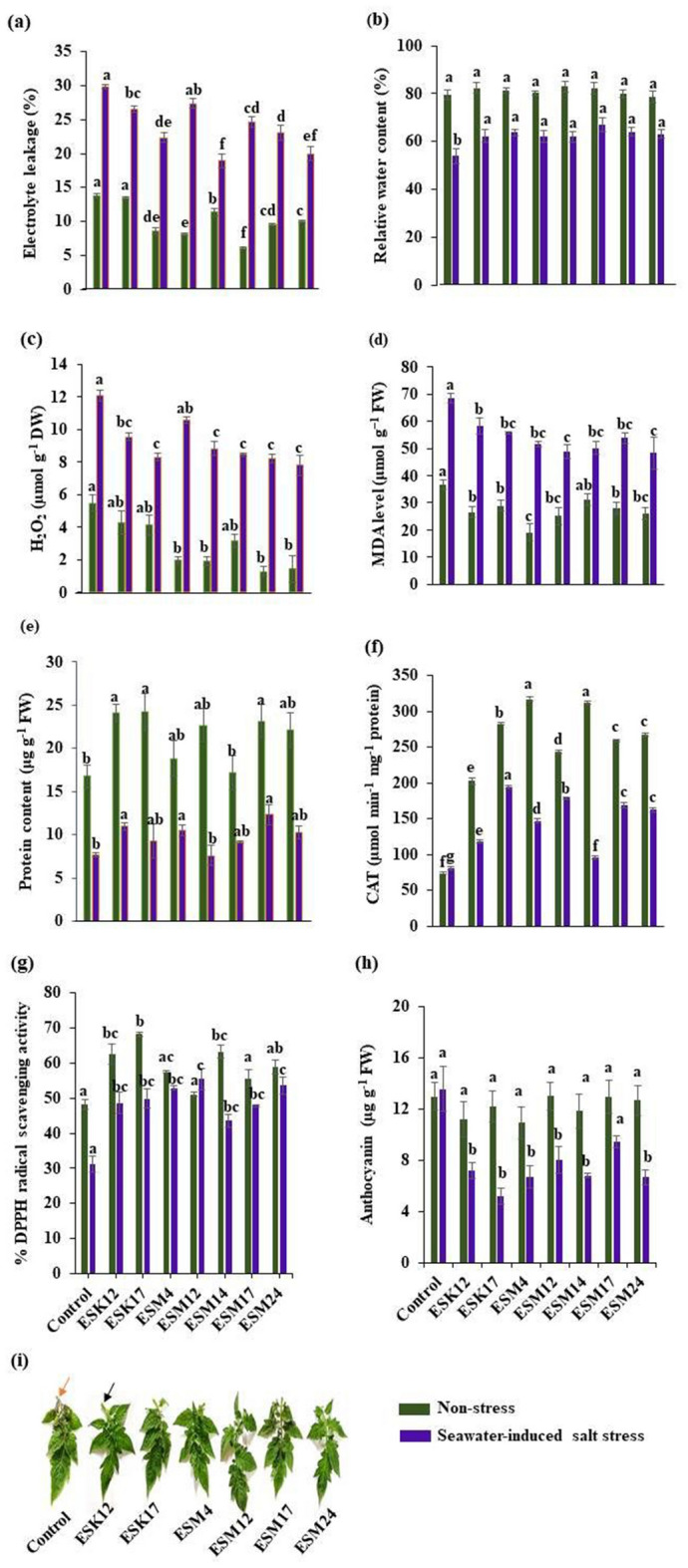
Halotolerant biofilm-producing rhizobacteria reduced sea water-induced salinity stress parameters and increased antioxidant defense systems in tomato plants. Pot-grown tomato plants were inoculated with different bacterial strains two times as described in “Methods” section. Analysis of (**a**) electrolyte leakage, (**b**) relative water content, (**c**) H_2_O_2_ production, (**d**) MDA level, (**e**) Protein content, (**f**) CAT activity, (**g**) % DPPH radical scavenging activity, (**h**) anthocyanin content, and (**i**) anthocyanin accumulation. Orange arrow-head indicate anthocyanin accumulation and black arrow-head indicate no accumulation. Green and blue bars in the graphs represent non-stress and seawater-induced salt stress. Values [mean ± standard error (SE)] of each treatment were attained from three biological replications (*n* = 3). Different letters represent significantly different data (P < 0.05 on Fisher’s least). *FW* fresh weight.

In order to detect the effects of biofilm-producing PGPR isolates to protect cells from oxidative damages, electrolyte leakage (EL) and relative water content (RWC) were measured. After 8 days of seawater-induced salt stress, all the bacterized plants exhibited lesser EL than the non-bacterized plants (Fig. 6a). Even under non-stressed conditions, bacterized plants showed a reduced EL. Among the tested PGPR, ESM12-inoculated plants showed the lowest EL (18.95%). The plants bacterized with ESM24, ESK17, ESM17, ESM14 or ESK12 diminished their EL by 19.99, 22.33, 23.07, 24.63 and 26.59%, respectively compared to the non-bacterized plants. However, ESM4 was not effective in reducing EL. Taken together, the association of PGPR with roots is an effective mechanism to protect from membrane leakage under normal and stressed conditions.

With an increased EL, the amount of RWC will be decreased. In this study, the leaf RWC was higher in all the PGPR-inoculated plants under seawater-induced salt stress conditions from 17 to 24% compared with the non-inoculated control plants (Fig. 6b). However, the RWC of the plants was not significantly affected by the inoculation of the PGPR isolates under non-stress conditions (Fig. 6b).

#### Accumulation of hydrogen peroxide and malondialdehyde

As an oxidative stress indicator, the accumulation of hydrogen peroxide (H_2_O_2_) and malondialdehyde (MDA) in the non-stress and seawater-induced salt stress plants was measured. The non-inoculated stressed plants accumulated 12.09 µmol g FW^−1^ H_2_O_2_, whereas PGPR-inoculated plants significantly showed reduced H_2_O_2_ levels under this condition. Among the PGPR isolates, ESK17, ESM12, ESM14, ESM17, and ESM24 lowered H_2_O_2_ levels by 30% (ca. 8.0 µmol g FW^−1^) compared to the non-inoculated control plants (Fig. 6c). ESK12 also significantly lowered the H_2_O_2_ (21%) production. In contrast, the 12% H_2_O_2_ reduction by ESM4 was insignificant. PGPR application also decreased H_2_O_2_ levels even in the non-stress plants. However, accumulation and the extent of H_2_O_2_ decrease in seawater-induced salt stress plants was twice as much as in non-stress plants.

ROS-oxidized membrane lipids generate MDA as a product of lipid peroxidation. Consistent with the reduction of H_2_O_2_, bacterized tomato plants also showed a reduced MDA accumulation under seawater-induced salt stress conditions (Fig. 6d). Although most of the PGPR isolates reduced MDA levels, ESM12 and ESM24 inoculation resulted in a more efficient reduction of MDA accumulation than in non-inoculated control plants. Bacterized non-stressed plants also exhibited reduced MDA levels. These suggest that biofilm-producing PGPR effectively reduces stress indicators even under normal growth conditions, though the indicators are increased in the seawater-induced salt stress conditions.

The protein content is also an indicator of the stress status of the plants. To prove the efficacy of biofilm-producing PGPR to reduce the seawater-induced salt stress, the total protein content was measured (Fig. 6e). The amount of total protein was significantly reduced in seawater-induced salt stress plants. All the bacterized plants reduced the degradation of total protein content except ESM12. ESM17, ESK12, and ESM4 decreased the protein degradation by 42, 30 and 26%, respectively, while ESK17, ESM14, and ESM24 reduced the total protein though insignificantly. Under non-stress conditions, bacterized plants also prevented the protein degradation, but it was not pronounced in the non-inoculated plants.

#### Antioxidants and radical-scavenging capacity of PGPR

Seawater-induced salt stress plants accumulated higher amounts of H_2_O_2_ and MDA (Fig. 6c,d). Thus, it is expected that free radical scavenging- and catalase (CAT) activity will also be increased in bacterized plants to scavenge H_2_O_2_ and MDA for preventing oxidative damage. Under seawater-induced salt stress, CAT activity was increased twofold in plants inoculated with ESK17, ESM12, ESM17, and ESM24 as compared to non-inoculated plants, and 1.5-fold with ESK12 and ESM4 (Fig. 6f). Under non-stress, bacterized plants also increased the CAT activity.

Total antioxidant capacity was determined in terms of %DPPH (1,1-diphenyl-2-picryl hydrazyl) radical-scavenging activity (Fig. 6g). Under seawater-induced salt stress conditions, non-inoculated tomato plants showed a 30% DPPH free radical decrease in their leaves. All the bacterized plants showed a significant reduction of %DPPH free radical from 48 to 55%.

Accumulation of anthocyanins in different parts of the plants is common under various adverse conditions. The antioxidant properties of the anthocyanins protect plants from various biotic and abiotic stresses including salt stress in plants^58^. In this study, non-inoculated seawater-induced salt stress plants accumulated the 13.53 µg anthocyanin g^−1^ FW. Except for ESM17, all the bacterized plants reduced (twofold) the anthocyanin contents (Fig. 6h). Anthocyanin accumulation was also visible in the leaves of the non-inoculated seawater-induced salt stress plants (Fig. 6i). Thus, biofilm-producing PGPR isolates are effective in attenuating the seawater-induced salt stress in tomatoes due to accumulation of anthocyanins.

#### Chlorophyll synthesis and biomass accumulation by application of PGPR

**Figure 7:**
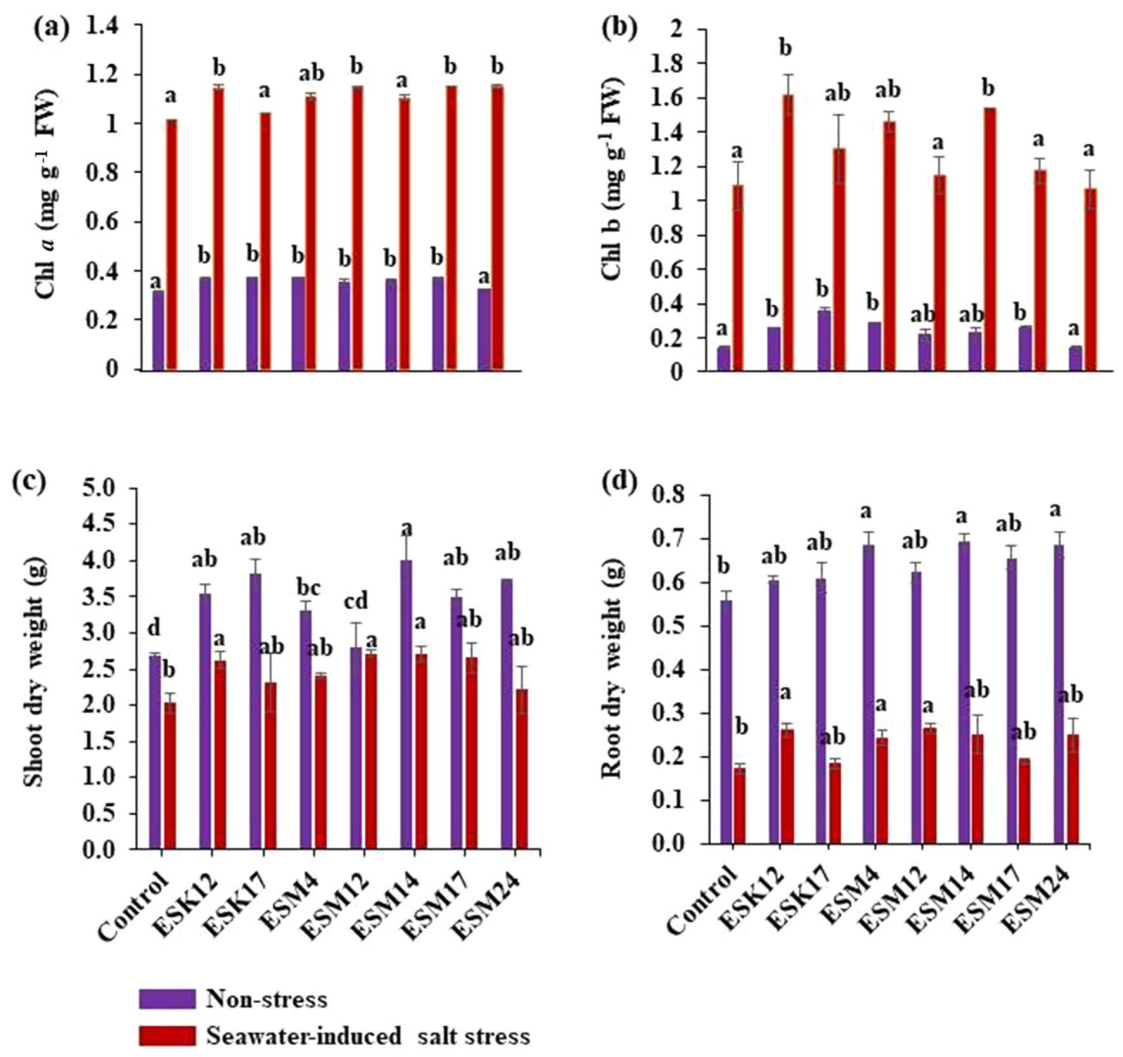
Effects of PGPR inoculation on the biomass accumulation and chlorophyll contents in tomato plants, grown under non-stress and seawater-induced salt stress conditions. Pot-grown tomato plants were inoculated with bacterial strains two times as described in “Methods” section. Analysis of (**a**) chlorophyll *a* content, (**b**) chlorophyll *b* content, (**c**) shoot dry weight, and (**d**) root dry weight. Blue and red bars in the graphs represent non-stress and seawater-induced salt stress. Values [mean ± standard error (SE)] of each treatment were attained from three biological replications (*n* = 3). Different letters represent significantly different data according to Fisher’s least significant difference (LSD) (P < 0.05). *FW* fresh weight.

Inoculation of halotolerant biofilm-producing rhizobacteria decreased the accumulation of H_2_O_2_ and MDA through an increased enzymatic activity under seawater-induced salt stress conditions in tomatoes (Fig. 6f–h). Hence, it is anticipated that the inoculation of different biofilm-producing rhizobacterial isolates will also increase chlorophyll (Chl) content and biomass accumulation in tomatoes. Non-inoculated tomato plants under seawater-induced salt stress conditions had 1.01 mg g^−1^ FW Chl *a*. ESK12, ESM12, ESM17, and ESM24 but not ESK17, ESM4, and ESM14 increased the production of Chl *a* (ca. 12%) compared to the non-inoculated control plants (Fig. 7a). Similarly, all the examined isolates also boosted the synthesis of Chl *b* (Fig. 7b). However, only ESK12- and ESM14-inoculated plants significantly increased (32% and 28%, respectively) Chl *b* synthesis and to a lower extent also for ESK17 and ESM4 with 13% and 22%, respectively.

In general, higher chlorophyll contents positively affect biomass accumulation in plants. In this study, ESK12- and ESM12-inoculated plants significantly increased the biomass accumulation in both roots and shoots under seawater-induced salt stress conditions compared to the non-inoculated control plant by about 30% (Fig. 7c) and 50% (Fig. 7d), respectively. ESM14- and ESM4-inoculated plants also showed increased biomass accumulation in both roots and shoots under seawater-induced salt stress conditions. On the other hand, ESK17- and ESM24-inoculated plants were only slightly increased in shoot biomass at the early growth stage of tomatoes (Fig. 7c,d). Under normal growing conditions, most of the bacterized plants were significantly increased in both root and shoot biomass compared to the non-bacterized plants. Thus, halotolerant biofilm-producing rhizobacteria effectively promote the growth of tomato plants under both non-stress and seawater-induced salt stress conditions.

#### Na^+^ biosorption and ionic balance in plants

**Figure 8:**
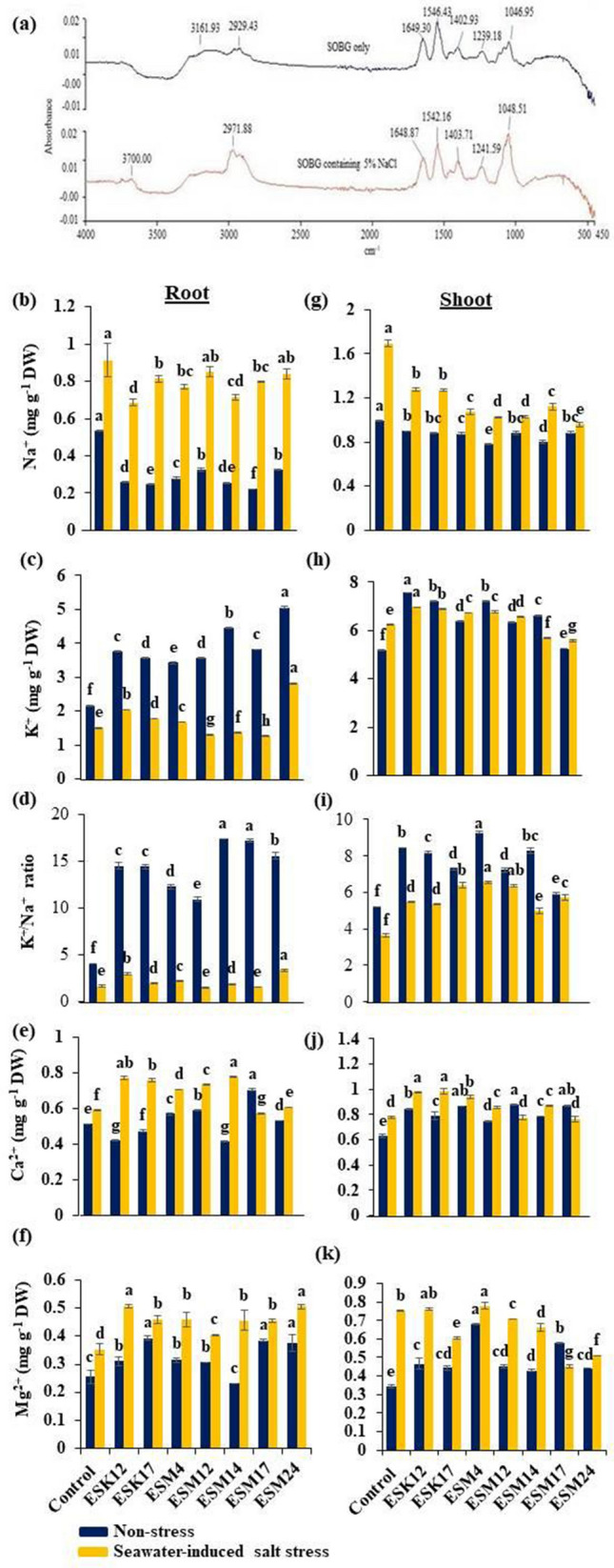
Shifting of peak positions in the biofilm biomass of *L. adecarboxylata* ESK12 after growth in SOBG containing 5% NaCl (lower lane) as compared to standard SOBG (upper lane) (**a**). Effects of the inoculation of halotolerant biofilm-producing rhizobacteria on ionic balance in roots and shoots of tomato plants. Pot-grown tomato plants were inoculated with different bacterial isolates. Na^+^, K^+^, K^+^/Na^+^, Ca^2+^, and Mg^2+^ were measured in the roots (**b–f**) (left) and shoots (**g–k**) (right). Blue and orange bars in the graphs represent the non-stress and seawater-induced salt stress. Values [mean ± standard error (SE)] of each treatment were attained from three biological replications (*n* = 3). Different letters represent statistically significant different data to Fisher’s least significant difference (LSD) (*P* < 0.001). *DW* dry weight.

To analyze how halotolerant biofilm-producing PGPR promoted plant growth and ameliorated seawater-induced salt stress damages, two representative bacterial isolates, namely ESK12 and ESM4 were inoculated in SOBG or SOBG containing 5% NaCl for biofilm formation. The biomass biofilm generated by these isolates was analyzed after centrifugation using FTIR. The peak positions of the biomass biofilm produced by ESK12 in SOBG containing 5% NaCl were shifted from 1046.95, 1239.18, 1402.93, 1546.43, 1649.30 and 2929.43 cm^−1^ to 1048.51, 1241.59, 1403.71, 1542.26, 1648.87 and 2971.88 cm^−1^, respectively as compared to the biomass biofilm produced by this bacterium in SOBG only (Fig. 8a). Several peaks in the biomass biofilm created in SOBG supplemented with 5% NaCl by ESM4 were also shifted when compared to the biomass biofilm generated in the SOBG only (data not shown). One explanation is that Na^+^ ions are absorbed by the various functional groups present in the bacterial biomass biofilm.

We then examined the effects of biofilm-producing PGPR isolates in maintaining the ionic balance in the roots and shoots of tomato plants. The inoculation of PGPR isolates significantly affected the accumulation of Na^+^, K^+^, Ca^2+^, and Mg^2+^ in the plant tissues (Fig. 8). In roots, all the bacterized non-stressed plants significantly lowered the Na^+^ content and increased the accumulation of K^+^. The isolates ESK12 and ESM14 lowered the accumulation of Na^+^ by 24% and 22% in roots, respectively (Fig. 8b). ESK17, ESM4, and ESM17 also effectively reduced the accumulation of Na^+^ in the roots (Fig. 8c–e). We found that ESM24 lessened Na^+^ accumulation by 43% in shoots, followed by ESM12 and ESM14 (Fig. 8g). ESM12 and ESK17 also increased K^+^ accumulation by 35% and 18%, respectively compared with the non-inoculated control plant. Surprisingly, ESM24 increased K^+^ accumulation by about 87%, while ESM4 to a lower extent. Importantly, bacterial isolates also maintained a higher K^+^/Na^+^ ratio under non-stress and seawater-induced salt stress conditions in roots (Fig. 8d). It is worthy that all of the bacterized plants under seawater-induced salt stress conditions increased the Ca^2+^ content by 15–35% and the Mg^2+^ content on an average by 50% in roots compared with the non-inoculated plants. In shoots, bacterized plants also maintained the ionic balance by a lowered accumulation of Na^+^ and increased accumulation of K^+^, Ca^2+^_,_ and Mg^2+^. Moreover, bacterized plants maintained a higher K^+^/Na^+^ ratio in both non-stress and seawater-induced salt stress conditions in shoots (Fig. 8i). As for shoots, the accumulation of K^+^, Ca^2+^, and Mg^2+^ were also showed an increasing trend (Fig. 8h–k). In general, the bacterized plants diminished Na^+^ accumulation and elevate K^+^ accumulation in both roots and shoots. These results suggest that halotolerant biofilm-producing PGPR especially ESK12, ESK17, ESM12, and ESM14 efficiently lowered the Na^+^ content and enhanced the K^+^, Ca^2+^ and Mg^2+^ content as well as the K^+^/Na^+^ ratio in the roots. Thus, these bacteria effectively retain ion homeostasis, which are essential for maintaining plant growth and developmental processes under seawater-induced salinity.

## Discussion

Forming a biofilm is one of the key salt-tolerant characteristics of rhizobacteria^39,40^. In this current study, 32.3% (21 of 65) halotolerant isolates formed biofilms. The identified genera, such as *Glutamicibacter*, *Enterobacter*, *Exiguobacterium, Staphylococcus*, *Leclercia*, *Pseudomonas* and *Bacillus*) were indeed reported as halotolerant bacteria^1,59,60^. The rhizobacteria isolated from peanut can tolerate 4–8% NaCl^60^. *Sphingobacterium* sp. BHU-AV3*, P. migulae* 8R6 and *P. fluorescens* YsS6 identified from tomato were shown to tolerate 0.85 M NaCl^61^. Recently, 74 rhizobacterial strains isolated from durum wheat were shown to tolerate 10% NaCl^62^. In our study, 94.74% and 73.68% of the isolates were able to tolerate 10% and 15% NaCl, respectively. All these identified bacteria also exhibited multiple plant growth-promoting (PGP) traits. Therefore, all these identified halotolerant biofilm-producing rhizobacteria are PGPR. Among the identified PGPR, *B. subtilis*^63^, *P. poae*^64^ and *P. putida*^65^ were formerly reported to form biofilms. Hence, *G. arilaitensis*, *G. nicotianae*, *E. ludwigii, E. cloacae*, *E. acetylicum*, *S. saprophyticus*, and *L. adecarboxylata* are novel halotolerant biofilm-producing PGPR identified in this study.

Secondary metabolites [including osmoprotectants or compatible solutes (e.g., proline and glycine betaine), volatile organic compounds (e.g., albuterol, 1,3-propanediol and acetoin) and extracellular polymeric substances (EPS)] produced by various halotolerant PGPR play a crucial role in ameliorating salinity stress^1,40^. Among the secondary metabolites, EPS contain hydroxyl, carboxyl and amino functional groups^36,37^. These functional groups were reported to ameliorate salt-stress in plants through binding with cations including Na^+^^39–41^. EPS also diminish the ion toxicity by minimizing the Na^+^ influx through an increased expression of the HKT1/K^+^ transporter in crop plants^66^. On the other hand, Dodd and Pérez-Alfocea^67^ have stated that EPS generate a rhizosheath around the plant’s roots, preventing the entry of Na^+^ into plants, thereby reducing salt stress. The rhizosheath was also shown to increase soil aggregation, water-holding capacity, and acquisition of nutrients (N, P, K and Fe) from soil, leading to increased plant growth^68^. EPS-producing *Halomonas* sp. EX01 and *Alcaligenes* sp. were demonstrated to increase osmotic stress tolerance and stimulate rice growth under salt-stress condition^69,70^. Furthermore, EPS-producing *G. halophytocola* KLBMP and *Pantoea alhagi* were reported to activate the antioxidant system, helping to withstand salt stress damage in crops^71,72^. Several studies also reported that PGPR producing IAA, ACC deaminase, catalase and acetoin alleviate the salinity stress in plants^1,29,60,73–75^. Our present study showed that the biofilm matrix and/or EPS contain several functional groups due to proteinacious curli, nanocellulose-rich polysaccharides lipids and nucleic acids. Our FTIR data also revealed that biomass biofilm absorbed the Na^+^ ions. Thus, the production of EPS, IAA, ACC, catalase and acetoin by PGPR might be one reason for alleviating the seawater-induced salt stress in tomatoes.

EL and RWC are two important parameters that determine the stress tolerance level in plants. Indeed, various abiotic stressors impair EL and RWC in plants. Among the abiotic stressors, salinity stress increases the EL^76,77^, but decreases the RWC^29,78–80^. A reduced RWC results in closing the stomata pore, diminishing photosynthesis, and accelerating senescence processes^81,82^. Moreover, depletion of RWC owing to salinity induces osmotic stress in plants^81^. Several studies have shown that PGPR-inoculated plants decreased the EL and increased the RWC in non-stress and under salt stress conditions^28,79,83–85^. Osmoprotactants (e.g., glycine betaine, proline, proteins and sugars) synthesized by different halotolerant PGPR strains have been shown to prevent membrane damage and water loss under salinity stress^46,83^. In the present study, bacterized plants considerably reduced the EL, and maintained a higher RWC than non-bacterized plants in seawater-induced salt stress conditions. In this study, all the non-inoculated plants synthesized significantly more proline than the bacterized plants in both non-stress and seawater-induced salt stress conditions (data not shown). The results of the study indicated that non-inoculated plants experience higher stress than bacterized plants because proline is one of the important stress marker^86^. However, all the bacterized plant leaves (except when inoculated with ESM12 under seawater-induced salt stress conditions) exhibited a significantly higher total protein content than the non-bacterized plants in both non-stress and seawater-induced salt stress conditions.

Plant cells accumulate ROS and degraded products of lipid peroxides [e.g. MDA, formaldehyde, acrolein, and 4-hydroxy-(E)-2-nonenal] which cause tissue damage during heat, drought, and salt stress^87,88^. To prevent the adverse effect of ROS, stressed plants induce an antioxidant defense system that scavenges the excess of ROS^7,18,23,87^. This study showed that seawater-treated non-inoculated plants accumulated higher levels of H_2_O_2_ and MDA, while bacterized plants effectively suppressed these levels. Accordingly, %DPPH radical scavenging activity, CAT activity and total protein production were dramatically increased in bacterized plants under seawater-induced salt stress conditions. Among them, the tetrameric heme-containing enzyme, CAT (E.C.1.11.1.6) is catalyzing H_2_O_2_^89^. Due to their antioxidant and iron binding properties, the total protein content plays a vital role in detoxifying ROS and free radicals, preventing lipid peroxidation^90^. The accumulation of anthocyanin in leaves improves plant adaptability to environmental stresses. Kitayama et al.^91^ have stated that plants exposed to salt stress synthesized higher amounts of anthocyanins, suggesting that plants suffer oxidative stress. In the present study, less anthocyanin accumulation was observed in bacterized plants than in non-bacterized plants under seawater-induced salt stress conditions. Recently, a higher antioxidant property was reported in EPS produced by halotolerant PGPR^40^. EPS produced by the halophytic endophyte *Glutamicibacter halophytocola* KLBMP prevented the oxidative damage in crops triggered by salinity^71^. Several PGPR secrete ACC deaminase that cleaves the ethylene precursor ACC (precursor of ethylene), thus lowering the concentrations of ethylene in stressed plants^46^. Indeed, PGPR producing ACC also enhanced salt tolerance and antioxidant enzyme activities, including CAT, in okra^75^. In this study, bacterized plants alleviated the seawater-induced salt stress in tomato by suppressing ROS levels and membrane lipid peroxidation. Bacterized plants also reduced the seawater-induced salt stress through a higher %DPPH radical scavenging- and CAT activity, total protein content, and anthocyanin synthesis. These mechanisms enable bacterized plants to promote chlorophyll synthesis and subsequently biomass accumulation. In the current study, ESK12, ESM4, ESM12 and ESM14 inoculation resulted in an increased biomass in both roots and shoots by 30–50% as compared to non-bacterized control plants under saline conditions. All the inoculated bacterial isolates efficiently colonized the roots in both non-stress (3.4E+9 to 4.6E+9 CFU g^−1^) and seawater-induced salt stress (3.6E+9 to 4.9E+9 CFU g^−1^) conditions (data not shown). Increased photosynthesis due to higher chlorophyll content results in higher photosynthates and finally in higher biomass accumulation. In this study, ESK12, ESM12, ESM17, and ESM24 significantly increased the chl *a* content and ESK12 and ESM14 the chl *b* content in tomato plants. Similar reports have shown that salt tolerant PGPR including *Achromobacter piechaudii* and *P. stutzeri* improved salt tolerance and increased the biomass accumulation of tomato seedlings under high salinity stress^92,93^. However, EPS enclosed with biofilms were not studied earlier to mitigate seawater-induced salt stress in tomatoes.

In response to salt stress, plants adjust ionic balance and cellular metabolism to continue growth and developmental processes^94,95^. In general, Na^+^ and Cl^−^ disturb ionic balance within the cells and adversely affect plant growth due to oxidative injury caused by ROS and lipid peroxidation^88^. The accumulation of Na^+^ within cells inhibits the absorption of K^+^ and translocation to the cellular compartment^44^. K^+^ is an essential element of various physiological processes in plants. It accelerates water movement and enzyme activation, which finally affects adenosine triphosphate (ATP) production and the rate of photosynthesis^96^. Na^+^ accumulation in soil solutions also prevents absorption of macronutrients, such as Ca^2+^ and Mg^2+^, which are essential for membrane stability and stomatal conductance^44^. Thus, increased accumulation of Na^+^ is toxic, but increasing accumulation of K^+^, Ca^2+^ and Mg^2+^ are beneficial for the various metabolic processes^96^. This study showed that seawater-induced salt stress provoked oxidative stress in plants and eventually suppressed plant growth. However, bacterized plants significantly exhibit a lowered accumulation of Na^+^ and ultimately maintained a higher K^+^ influx in shoot under seawater-induced salt stress conditions. Notably, bacterized tomato plants also maintained a higher K^+^/Na^+^ ratio in the roots and shoots. The approximation of Ca^2+^ and Mg^2+^ content in root and shoot biomass also showed that halotolerant biofilm-producing rhizobacteria increased their accumulation in both non-stress and seawater-induced salt stress plants. Thus, halotolerant biofilm-producing rhizobacteria maintain the ionic balance and ion homeostasis by decreasing the accumulation of harmful Na^+^ and increasing the accumulation of K^+^, Ca^2+^ and Mg^2+^. In nature, 99% of bacteria form biofilms on different biotic and abiotic surfaces. In this study, Na^+^ is bound by the biomass biofilm. Thus, it is believed that Na^+^ bound by the bacterial biofilm formed around the roots by various halotolerant biofilm-producing bacteria, prevents Na^+^ to enter into the cellular compartment. On the other hand, the absorption of K^+^ and macro-elements including Ca^2+^ and Mg^2+^ are instead increased by the bacterization of tomatoes.

This study extends our understanding that halotolerant biofilm-producing rhizobacteria can enhance crop productivity under saline agro-ecosystems. The experimental results showed that the identified halotolerant biofilm-producing bacteria attenuated seawater-induced salt stress and enhanced biomass accumulation in tomato plants through the formation of biofilms, availability of nutrients (e.g. N, P, Fe, and K), production of IAA, ACC deaminase and NH_3_, expression of antioxidant defense systems, and maintenance of a proper ionic balance. Unfortunately, pure culture isolates with multiple PGP traits does not always enhance plant growth under field conditions, due to a competition with indigenous microflora and other environmental stresses. Other challenges that affect survival and colonization in roots under field conditions by PGPR are inappropriate carrier used for bio-formulation, lower numbers of CFU, lower shelf-life of the inoculants, and poor soil health owing to soil pollution and long-term fumigation. Still, we believe that halotolerant biofilm-producing bacteria with multiple PGP activities would be a beneficial approach in addressing the above challenges.

## Methods

### Isolation of halotolerant biofilm-producing rhizobacteria

Tomato plants (*Solanum lycopersicum* L.) grown in the saline (3.5–5.0 dS m^−1^) affected areas, such as Patuakhali and Bagerhat district, Bangladesh were carefully uprooted during the vegetative stage. The rhizosphere samples were prepared as described by Haque et al.^64^. Each rhizosphere sample was then homogenized with sterile 0.5 × phosphate buffer saline (PBS) using a sterile mortar and pestle. Halotolerant rhizobacteria were isolated as explained by Jha et al.^97^ with minor modifications. In brief, 50 µL diluted sample was inoculated in glass test tubes containing 5 mL yeast extract peptone [YEP peptone (1%), yeast extract (0.5%), pH 6.8)] broth containing 5% NaCl instead of nitrogen-free semisolid NFb media containing malate and 4% NaCl. The inoculated test tubes were kept at 28 °C in a shaking condition (160 rpm). After a 72-h incubation, 100 µL suspension was transferred onto YEP agar (1.5%) plates (3 plates sample^−1^) supplemented with 5% NaCl. Colonies formed on the plates after 48 h incubation at 28 °C were herein termed as halotolerant rhizobacteria. Morphologically diverse (e.g., size, shape and pigmentation) and well separated from others, five colonies (isolates) from each sample were picked using sterile toothpicks, and sub-cultured to prepare a pure culture. All the halotolerant rhizobacterial isolates were screened for biofilm formation as described by Haque et al.^98^. In brief, each isolate was initially cultured in YEP broth overnight. Then, 50 μL culture (ca. 10^6^ CFU mL^−1^) was injected the flat bottom glass test tubes (Pyrex, UK) containing salt-optimized broth [SOB (for 1 L: 5 g yeast extract, 20 g tryptone, 2.4 g magnesium sulphate, 0.5 g sodium chloride, and 0.186 g potassium chloride)] containing 2% glycerol (SOBG) media. The test tubes were set at 28 °C without shaking. The AL (air–liquid) and SAL (solid-air–liquid) biofilms were categorized and pictures were taken. To quantify the formation of biomass biofilm, 72-h old biofilm matrices were carefully transferred into 15 mL falcon tubes and centrifuged (15,000 rpm for 10 min). After centrifugation, each pellet (biofilm matrix) was weighed using a digital balance. The CFU from biofilm matrices was quantified as explained^99^.

### Identification of bacteria

Bacterial genomic DNAs were isolated using a standard protocol^100^. The 16S rRNA gene was amplified using 27F (5'-AGAGTTTGATCMTGGCTCAG-3') and 1492R (5'-GGTTACCTGTTACGACTT-3') primers by PCR. For sequencing, 3500 Genetic Analyzer (Applied Biosystems) was used. Gene sequences of different bacterial isolates were compared against the sequences of bacteria available in NCBI data banks (http://www.ncbi.nlm.nih.gov/) using the Basic Local Alignment Search for Nucleotide (BLASTN) program. The obtained 16S rRNA gene sequences were deposited in the GenBank nucleotide databases (http://www.ncbi.nlm.nih.gov/Blast.cgi). Using 16S rRNA gene sequence data, a phylogenetic tree was constructed. MUSCLE alignment and maximum likelihood method were used to construct the tree with gBlock used for alignment refinement.

### Identification of the biofilm matrix components

In order to identify the biomolecules and chemical functional groups/ligands present in the biofilm matrices/EPS, the FTIR spectroscopy was used. Initially, biofilm matrices were harvested by centrifugation (12,000 rpm for 10 min) to discard the liquid. The pellets were further analyzed. To identify the components, 450–4000 cm^−1^ was scanned (16 scans at 4 cm^−1^ resolution and at 0.2 cm s^−1^ scanning speed) using the TGS (Triglycine sulphate) detector. The IR spectra of the biofilm matrices were acquired using the Perkin Elmer FTIR (Spectrum-2) instrument and the CPU32M software. To analyze the baseline subtracted biofilm spectra, Perkin Elmer’s proprietary software (Version 10.05.03) was used. Curli and/or cellulose production were checked by Congo red- and Calcofluor binding assays^99^. In this case, 2 μL culture (overnight grown) was dotted onto the agar plates supplemented either Congo red (40 μg mL^−1^) or Calcofluor white (200 μg mL^−1^). The plates were then kept at 28 °C for 48 h. Calcofluor binding (but not the Congo red binding) was monitored under UV light (366 nm) and photos taken. Nanocellulose fibers present in the bacterial biofilm matrices were observed by SEM (JEOL JSM-6490LA, Japan). For SEM analysis, 72-h old biofilm matrices were carefully collected and centrifuged at 10,000 rpm for 10 min. The pellets were gently oven-dried (50 °C for 24 h)^33^. Each pellet was fixed on adhesive carbon tape and coated with platinum using a vacuum coater. The images were taken by SEM operated at 5.0 kV.

### Preparation of inoculum for plant growth-promoting tests

A single colony of each isolate was injected in the glass test tube containing YEP broth and incubated at 28 °C in agitated condition (160 rpm) until optical density (OD_660_) reached 0.6–0.8. Each culture (1 mL) was then centrifuged at 12,000 rpm for 10 min. Each pellet was re-suspended in YEP broth. In order to bioassays of the in vitro PGP activities, 2 µL (ca. 10^7^ CFU mL^−1^) culture was spotted onto agar plates (4 dots plate^−1^), while 50 µL culture was inoculated in YEP broth.

### In vitro plant growth promoting tests

All the PGP tests were performed using the respective medium (as mentioned below) supplemented with 5% NaCl. IAA production was quantified using the Salkowsky colorimetric- and HPLC method as described earlier by Gordon and Weber^101^ and Myo et al.^102^, respectively. In both the cases, all the biofilm-producing isolates were grown in LB (Luria Bertani) broth supplemented with L-tryptophan (0.2%) in agitated condition. For colorimetric determination, Salkowsky reagent (35% perchloric acid and 0.5 M FeCl_3_) was mixed with each culture supernatant and kept at room temperature in dark. After 45 min, IAA (an auxin) was estimated by spectrophotometer with an absorbance at 530 nm. Different known concentrations (0–1000 μg mL^−1^) of IAA (Sigma-Aldrich, St. Louis, MO, USA) were used as standard. For HPLC analysis, cell-free extract was acidified then the organic phase was collected. After evaporation of ethyl acetate, HPLC-grade methanol was added to dissolve the crude extract. Shimadzu Prominence HPLC (Japan) with a C18 analytical column (4.6 mm × 250 mm, 5 μm) were used to quantify IAA. Methanol and 1% acetic acid (50:50 v/v) were used as the mobile phase. Column temperature, flow rate and injection volume were maintained at 25 °C, 1 mL min^–1^ and 20 μL, respectively. Lab Solutions software was used to evaluate the data, comparing it with the elution profiles of standard IAA.

Nitrogen fixation study was done according to Ker^103^. In addition, two forward primers, F1-TAYGGIAARGGIGGIATYGGIAARTC and F2-TGYGAYCCIAAIGCIGA, and one reverse primer, R6-TCIGGI GARATGATGGC were used to amplify the 470 bp and 359 bp *nifH* fragment by PCR^104^ from each bacterial genomic DNA. PCR conditions with 100 ng template DNA were as follows: one cycle at 95 °C for 5 min, 40 cycles at 95 °C for 30 s, 51 °C for 30 s and 72 °C for 1 min plus one cycle at 72 °C for 7 min. For P solubilization, 2 µL suspension was dotted onto National Botanical Research Institute’s Phosphate (NBRIP) agar plates [for 1 L: glucose 10 g, magnesium chloride (MgCl_2_.6H_2_O) 5 g, magnesium sulphate (MgSO_4_.7H_2_O) 0.25 g, potassium chloride (KCl) 0.2 g, ammonium sulphate [(NH_4_)_2_SO_4_] 0.1 g, and agar 15 g] containing 0.5% tricalcium phosphate [Ca_3_(PO_4_)_2_]^105^ or 0.8% rock phosphate^64^. The P solubilization index (PSI) was also calculated^64^. For K solubilization, 2 µL culture of each isolate was dotted onto Aleksandrov agar plates [for 1 L: glucose 5.0 g, MgSO_4_·7H_2_O 0.5 g, FeCl_3_ 0.005 g, CaCO_3_ 0.1 g, Ca_3_(PO_4_)_2_ 2 g, AlKO_6_Si_2_ (potassium aluminum silicate) 2 g, and agar 15 g]^106^. AlKO_6_Si_2_ was used as a source of insoluble inorganic potassium. For Zn solubilization, 2 µL suspension of each bacterium was dotted onto basal agar plates (per 1 L: glucose 10 g, (NH_4_)_2_SO_4_ 1 g, KCl 0.2 g, K_2_HPO_4_ 0.1 g, MgSO_4_·7H_2_O, and agar 15 g, pH adjusted to 7.0) containing 0.2% insoluble Zn^106^. In this study, Zn_3_(PO_4_)_2,_ ZnCO_3_, and ZnO (Wako Pure Chemical Industries Ltd., Japan) were used as insoluble Zn sources. All the spotted plates (P, K, or Zn solubilization) were placed at 28 °C. Development of a clear zone surrounding the colonies within 7 days suggested a positive P, K, or Zn solubilization. In order to detect the production of siderophore i.e., Fe solubilization, 2 µL culture of each isolate was dotted onto the LB agar plate and incubated. After 24 h, 8 mL O-CAS broth^107^ was applied over those LB agar plates. The color change from blue to light yellow, yellow, light orange/orange or purple indicated a positive siderophore production. Based on color, the siderophore was classified as catechol (purple), hydroxamate (light orange/orange), carboxylate (light yellow), and mixture of hydroxamate- and carboxylate (yellow)^57^.

Qualitative production of volatile components including acetoin, indole, NH_3_ and HCN were determined as described by Dye^108^, Lelliott and Dickey^109^, Dinesh et al.^110^, and Lorck^111^, respectively. For acetoin production, 30 μL bacterial culture was mixed with 3 mL broth of yeast extract salt and incubated under shaking condition. After 72 h, 500 μL culture was mixed with 300 μL α-napthol (5%) in absolute alcohol (v/v). Formation of a crimson to ruby color on the surface within 5 h indicated a positive acetoin production. For indole production, 30 μL bacterial suspension was injected in 3 mL broth containing tryptone (10 g L^−1^), l-tryptophan (1 g L^−1^) and the required amount of water (L^−1^) and the tubes were placed at 28 °C in 160 rpm. After 3 days, Kovac’s reagent (250 μL) was added and mixed gently. Formation of a dark red color on the surface of the mixture indicated a positive indole production. For NH_3_ production, 30 μL culture was suspended in the tube containing 2.5 mL peptone water and kept at 28 °C with 160 rpm. After 3 days, Nessler’s reagent (500 μL) was added to each test tube. Yellow to brown color formation confirmed a positive NH_3_ production. In case of HCN production, a single colony of each bacterium was streaked on YEP plates supplemented with glycine (0.45%). Then, three sterilized filter papers were soaked in picric acid (0.25%) and sodium carbonate (1.25%) solution and placed on the lids of petri plates. The plates were then sealed and kept at 28 °C for 24 h. The color change of the filter papers from greenish to yellow, reddish or brown indicated a positive HCN production.

The rhizobacterial isolates were screened for ACC deaminase activity on the sterile minimal DF (Dworkin and Foster) salts media^112^ supplemented with 3 mM ACC (1-cyclopropoane-1-carboxylic acid) as the sole nitrogen source^113^. In brief, each isolate (50 μL) was plated on minimal Dworkin and Foster (DF) salt agar plates. A 50 μL suspension was also plated onto ACC-deficient minimal DF salt agar plates as a negative control. The plates were incubated at 28 °C for 5 days. Colonies formed on the plates were considered as ACC-deaminase producers. Catalase, oxidase, gelatinase, and arginine dihydrolase were also qualitatively determined as described in Hayward^114^, Shekhawat et al.^115^, Schaad^116^, and Thornley^117^, respectively, while lipase^110^, cellulase, and protease assays were carried out as described^118,119^. For catalase production, a loop-full (24-h old) culture was placed on a clean glass slide then a drop of 3% H_2_O_2_ was added. Production of gas bubbles indicated a positive reaction. For oxidase test, a strip of filter paper was soaked with 3 drops of N,N,Nˊ,Nˊ-Tetramethyl-p-phenylenediamine dihydrochloride (color indicator). Then, a loop-full overnight-grown culture of each isolate was smeared onto the surface of the filter paper. Development of a purple color within 10 s indicated a positive reaction. For gelatinase production, a medium (1 L) containing beef extract (3 g), gelatin (120 g), peptone (5 g) and an adequate amount of water was prepared. All ingredients were dissolved by steam heating, then 3 mL suspension was poured in test tubes and autoclaved. After cooling the tube, stab inoculation was done for each bacterium and the tubes placed at 28 °C. After 72 h, the test tubes were transferred to 4 °C for 30 min. A flow of the medium after gently tipping indicated a positive gelatinase production. In case of lipase production, 1 μL suspension of each isolate was dotted onto YEP agar plates (4 dots plate^−1^) containing Tween 20 (1%). Development of translucent zones around colonies within 48 h incubation at 28 °C confirmed a positive lipase production. For cellulase production, 1 µL suspension of each isolate was marked onto LB agar plates supplemented with carboxymethyl cellulose (1%) and placed at 28 °C. After 24 h, the plates were stained with 0.01% Congo red for 15 min, followed by de-staining with 1 M NaCl. Formation of yellowish zones around bacterial colonies indicated positive results. In case of protease production, 1 µL suspension was dotted onto LB agar plates containing 1% skim milk and incubated at 28°C. Formation of clear zones around colonies within 3 days suggested a positive protease production. In all the cases, *Pseudomonas chlororaphis* ESR15^64^ was used as a positive control, while *Bacillus cereus* ESD3^43^ was only used as a positive control for the protease test. All these experiments were repeated three times with 4 replications.

### Abiotic stress tolerance tests

All the isolates were confirmed for their ability to grow on agar plates at different concentrations of NaCl (15, 10 and 5%), temperatures (50, 42 and 37 °C) and varying pHs (10, 9, 8, 7 and 4). In all these assays, a 50-µL aliquot was plated on LB agar plates. In order to examine the effect of NaCl and pH, the plates were incubated at 28 °C. In contrast, the plates were incubated at the desired temperatures to study the effect of different temperatures on growth. In all cases, the growth was examined after 96 h of incubation. *B. cereus* ESD3 was used as a positive control^43^. All tests were performed twice with three replications.

### In vitro antagonistic activities

All the halotolerant isolates were further tested for their antagonistic activities against *X. campestris* pv. *campestris* ATCC 33913 (causal agent of bacterial leaf spot in tomato), *R. solanacearum* ATCC® 11696™ (causal agent of wilt in tomato), and *P. carotovorum* subsp. *carotovorum* PCC8 (causal agent of soft rot in tomato, accession number KX098362) as described by Furuya et al.^120^. In brief, each rhizobacterial isolate (1 μL) was dotted onto YEP agar plate and kept for 48 h. A sterilized filter paper soaked in chloroform was placed in the lid of the Petri dish to kill the rhizobacterium. The petri dishes were placed at room temperature for 1.5 h. Then, melted water agar (50 °C) containing ca. 10^9^ CFU mL^–1^ plant pathogenic bacterium was decanted onto the plates and incubated at 28 °C. Development of an inhibition zone nearby the growth region of the rhizobacterium within 48 h indicated a positive biocontrol agent.

### Setting of pot experiment

BARI (Bangladesh Agricultural Research Institute) Tomato 2 was used in this study. Seedlings were grown according to the method described in Haque et al.^64^. In brief, healthy seeds were surface-sterilized using 5% NaOCl followed by 4 times washing with sterile distilled water. Then, the seeds were sown in a plastic tray containing sterile sand. For pot experiment, 3 parts of sterilized vermicompost were mixed with 1 part of sterilized garden soil (1.7% organic matter, 0.09% total N, 1.75 mg kg^−1^ total P and 98.6 mg kg^−1^ exchangeable K) and then each pot (size = height × diameter = 15 cm × 50 cm) was filled with 2 kg soil (soil-vermicompost mixture).

### Experimental design and treatments

The experiment was conducted following a completely randomized design (CRD) with 8 treatments and 4 replications. The treatments were (i) without inoculation of PGPR i.e., control; (ii) *Leclercia adecarboxylata* ESK12; (iii) *Enterobacter ludwigii* ESK17; (iv) *Glutamicibacter arilaitensis* ESM4; (v) *E. cloacae* ESM12; (vi) *B. subtilis* ESM14; (vii) *P. putida* ESM17; and (viii) *Exiguobacterium acetylicum* ESM24. Plant growth promotion was evaluated after the application of these treatments in both non-stressed (sterile tap water used as irrigation) and seawater-induced salt-stress (sterile seawater used as irrigation) conditions. Novel biofilm-producing PGPR strains with no previous report of plant growth promotion under seawater-induced salt stress were used in this study.

### Root colonization and imposing seawater-induced salt stress

Each PGPR strain as mentioned above was grown in LB broth overnight under shaking condition and then diluted to ca. 10^8^ CFU mL^−1^. Uniform size seedlings (16 days old) were uprooted, and the root zones washed with sterile distilled water. Then, root bacterization was performed twice: during transplanting (16 day old seedlings) and after 7 days of transplanting as described by Haque et al.^64^. Afterwards, one seedling was transferred per pot and kept under poly shade for growth. For non-stress, 24-day old plants were irrigated with only sterile tap water, while sterile seawater with 10 dS m^−1^ salinity was applied as irrigation for seawater-induced salt stress. In this study, the soil salinity was achieved at 10 dS m^−1^ within 5-times of irrigation. In both types of stresses, irrigation was done to field capacity [when water was leached through bottom holes of the pots, this was considered maximum field capacity] at one-day-interval. Leaves of 40-day-old plants were collected to determine physiochemical changes and biomass accumulation in plants. This experiment was repeated two times. This pot experiment was conducted during winter season (November to December in 2021). The average air temperature in November and December was 24.58 °C (maximum 34.5 °C and minimum 13.5 °C) and 19.51 °C (maximum 30.0 °C and minimum 8.0 °C), respectively. The average relative humidity in November and December was 87.06 and 87.0% respectively. The total amount of rainfall in November was 2.92 mm whereas no rainfall was observed during December (https://bsmrau.edu.bd/age/weather-data/).

### Electrolyte leakage

Leaf discs of the third leaf from the top were used to determine electrolyte leakage (EL). Briefly, 1 cm^2^ of 5 leaf discs were transferred into glass tubes containing 10 mL distilled water. The electrical conductivity (EC) of the leaf sample was measured at room temperature twice after incubation in water bath at 40 °C and 100 °C for 30 min, respectively^121^. Then, the EL was calculated using the following formula:

$${\text{EL }}\left( \% \right) \, = \, \left[ {\left( {{\text{EC at 4}}0 \, ^\circ {\text{C }}{-}{\text{ EC at 1}}00 \, ^\circ {\text{C}}} \right)/{\text{EC at 1}}00 \, ^\circ {\text{C}}} \right] \, \times { 1}00$$

### Relative water content

Five leaf discs (1 cm^2^) from the top third leaf were taken and immediately the fresh weight was measured to determine the relative water content (RWC). The turgid weight of the leaf discs was measured after incubation in distilled water at 4 °C for 24 h. Then the leaf discs were oven-dried at 72 °C for 48 h and the dried weight was recorded. The RWC was calculated as the following formula:

$${\text{RWC }}\left( \% \right) \, = \, \left[ {\left( {{\text{Fresh weight }}{-}{\text{ Dry weight}}} \right)/\left( {{\text{Turgid weight }}{-}{\text{ Dry weight}}} \right)} \right] \, \times { 1}00$$

### Quantification of hydrogen peroxide levels

Hydrogen peroxide (H_2_O_2_) concentration was determined according to Loreto and Velikova^122^. For this, 0.5 g leaf samples were homogenized in 3 ml 1% (w/v) tri-chloroacetic acid (TCA). The homogenate was centrifuged at 10,000 rpm for 10 min at 4 °C. Subsequently, 0.75 mL of the supernatant was added to 0.75 mL of 10 mM phosphate buffer (pH 7) and 1.5 mL of 1 M KI. The H_2_O_2_ concentration of the supernatant was evaluated by comparing its absorbance at 390 nm to a standard calibration curve. The concentration of H_2_O_2_ was calculated from the standard curve, and the concentration expressed as µmol g^−1^ FW.

### Quantification of lipid peroxidation

The lipid peroxidation level was quantified by the thiobarbituric acid (TBA) reaction as described by Vemanna et al.^123^. The representative lipid peroxidation product malondialdehyde (MDA) was quantified from bacterized and non-bacterized leaf samples of plants from both non-stress and seawater-induced salt stress conditions. Fresh leaf samples (0.2 g) were ground in 2.5 mL of 0.1% trichloroacetic acid (TCA) and centrifuged at 14,000 rpm for 15 min. After centrifugation, 1 mL of the supernatant was mixed with 2.5 mL 0.5% TBA in 20% TCA and incubated in a hot water bath (95 °C) for 30 min. Thereafter, the reaction was stopped immediately on ice and then centrifuged at 10,000 rpm for 10 min. Absorbance at 532 nm and 600 nm was determined, and MDA concentration was estimated by subtracting the non-specific absorption at 600 nm from the absorption at 532 nm using an absorbance coefficient of extinction (155 mM^−1^ cm^−1^).

### Measurement of leaf anthocyanin contents

Leaf anthocyanin contents were spectrophotometrically estimated by differential pH method. For this, leaf samples (0.2 mg) were ground with 2 mL extraction buffer (methanol:water:concentrated HCL solution; 80:20:1 v/v/v). After centrifugation at 14,000 rpm, 0.4 mL supernatant was mixed in a separate tube with 3.6 mL of potassium chloride buffer (pH 1.0, 0.025 M) and sodium acetate buffer (pH 4.5, 0.4 M). Then the absorbance was recorded at 510 nm and 700 nm, respectively and water was used as blank^124^. Mean absorbance (A) of the two buffers was calculated as follows:

$${\text{A }} = \, \left( {{\text{A}}_{{{51}0}} {-}{\text{ A}}_{{{7}00}} } \right){\text{ pH 1}}.0 \, {-} \, \left( {{\text{A}}_{{{51}0}} {-}{\text{ A}}_{{{7}00}} } \right){\text{ pH 4}}.{5}$$

Total anthocyanin content was estimated using the following equation:

$${\text{Anthocyanin }}\left( {{\text{mg g}}^{{ - {1}}} {\text{FW}}} \right) \, = \, \left( {{\text{A }} \times {\text{ MW }} \times {\text{ DF }} \times { 1}000} \right)/\varepsilon \times {1}$$

where A is the absorbance of the sample, DF is the dilution factor (10), MW is the molecular weight of cyaniding-3-glucoside (449.2), a major anthocyanin, ε is the molar extinction coefficient of cyaniding-3-glucoside (26,900 in L × mol^–1^ cm^–1^) and 1 is the path length (1 cm).

### Quantification of catalase activity

Total protein was first extracted from the leaf sample to measure the catalase (CAT) activity. About 0.2 mg leaf sample was homogenized in phosphate buffer (50 mM, pH 7.0) containing potassium chloride (100 mM), ascorbate (1 mM), *β*-mercaptoethanol (5 mM) and glycerol (10%, v/v). Then, the homogenized sample was centrifuged at 14,000 rpm for 15 min at 4 °C. One mL reaction mixture was prepared for phosphate buffer (100 mM, pH 7.2), EDTA (0.1 µM), and H_2_O_2_ (0.1%). The reaction was started after adding 20 µg of protein and then decrease of H_2_O_2_ was monitored at 240 nm with a spectrophotometer (APEL PD-303, Japan). CAT activity was expressed as µmol min^−1^ mg^−1^ protein.

### %DPPH radical scavenging activity

The total antioxidant activity of the leaf samples was estimated by the radical scavenging activity using the 1,1-diphenyl-2-picryl hydrazyl (DPPH) method^125^. Briefly, 0.2 g leaf was crushed in phosphate buffer (100 mM, pH 7.0). After centrifugation at 14,000 rpm for 15 min at 4 °C, 0.1 mL supernatant was added to the DPPH solution (0.9 mL of 0.2 mM DPPH in ethanol). Then, the reaction mixture was incubated at 25 °C for 30 min in dark, after which the absorbance was measured at 517 nm. Instead of plant extract, the equal amount of methanol was added to DPPH solution as a control. The percent DPPH radical scavenging activity was estimated as follows:

$$\% {\text{ scavenging of DPPH }} = \, \left[ {\left( {{\text{A}}_{0} {-}{\text{ A}}_{{1}} } \right)/{\text{A}}_{0} } \right] \, \times { 1}00$$

where A_0_ is the absorbance of the control and A_1_ is the absorbance of the plant extracts.

### Measurement of chlorophyll content

Chlorophyll (Chl) contents were extracted from 0.2 g of leaf tissues in 5 mL of acetone (90%) and then kept at room temperature for 48-h in the dark. The supernatant was topped to a final volume of 10 mL. The absorbance was recorded at 663 nm and 645 nm using a UV–visible spectrophotometer. Chl *a* and Chl *b* were estimated by using the following formula and expressed as mg g^−1^ FW^51^.

$$\begin{gathered} {\text{Chl}}\ a = \, \left[ {{12}.{7 }\left( {{\text{A}}_{{{663}}} } \right) \, {-}{ 2}.{69 }\left( {{\text{A}}_{{{645}}} } \right)} \right] \, [{\text{V}}/\left( {{1}000 \, \times {\text{W}}} \right] \hfill \\ {\text{Chl}}\ b = \, \left[ {{22}.{9 }\left( {{\text{A}}_{{{645}}} } \right) \, {-}{ 4}.{68 }\left( {{\text{A}}_{{{663}}} } \right)} \right] \, [{\text{V}}/\left( {{1}000 \, \times {\text{W}}} \right] \hfill \\ \end{gathered}$$

### Estimation of biomass accumulation

Forty-day-old plants were harvested from the pots to quantify biomass accumulation in the roots as well as in the shoots. The root-adhering soils were carefully removed by gentle washing with running tap water. Then the roots and shoots were separated and kept in brown paper for oven drying at 70 °C until a constant weight was achieved. Dry weight was recorded to calculate the biomass accumulation in plants.

### Quantification of Na^+^, K^+^, Mg^2+^ and Ca^2+^ from tomato plants

Na^+^, K^+^, Mg^2+^ and Ca^2+^ were quantified from dried root and shoot samples of tomato plants grown under non-stress and seawater-induced salt stress conditions using an Inductively Coupled Plasma Atomic Emission Spectrometer (ICP-AES, ICP9820, Shimadzu, Japan.) Microwave sample digestion was carried out at 1200 MW with ETHOS EASY (Milestone, USA) instrument. The samples were digested using the program recommended by the manufacturer (14 vessels). The calibration solutions were prepared by appropriate dilutions of multi-element standard solution 1 for ICP, Ca^2+^ and Mg^2+^ both 10 mg L^−1^, Na^+^ and K^+^ 50 mg L^−1^ and 100 mg L^−1^, respectively made by Sigma-Aldrich. (TraceCERT, CRM 90243). For the calibration curve, four points were set at 0.0, 0.5, 1.0, 1.5 ppb for Ca^2+^ (r = 0.999), Mg^2+^ (r = 0.999) and Na^+^ (r = 0.999); 0.0, 5.0. 10.0, and 15.0 ppb for K^+^ (r = 0.999). Suprapure 69% HNO_3_ (m/m, Sigma Aldrich, USA) and deionized water with the maximum resistivity of 18.2 MΩ cm^−1^ (Direct-Q UV water purification system) were used for sample (pre-) treatment and sample dilution.

### Statistical analysis

A one-way analysis of variance (ANOVA) test was done for the in vitro experiments, while multiple factor ANOVA test was performed for the in vivo pot experiments. In all the cases, Shapiro–Wilk test was used to confirm the normal distribution of the data sets. ANOVA, distribution of data, homogeneity of variance, and mean comparison of treatment effects were analyzed using the R software version 3.3.6. Fisher’s least significant difference test was applied to compare the means.

### Ethical statement

Experimental research and pot experiments in this study were conducted as per guidelines and recommendation of ‘Biosafety Guidelines of Bangladesh’ published by Ministry of Environment and Forest, Government of the People’s Republic of Bangladesh (2005). The released variety, BARI (Bangladesh Agricultural Research Institute) Tomato 2 used in this study was collected from Bangladesh Agricultural Research Institute, Gazipur-1701, Bangladesh. Three researchers (KN, MMI, and FEE) from Bangladesh Agricultural Research Institute worked on this manuscript.

## Supplementary Information

Supplementary Figure 1.

Supplementary Figure 2.

Supplementary Figure 3.

Supplementary Figure 4.

Supplementary Table 1.
