# Supplementary material for: Halotolerant biofilm-producing rhizobacteria mitigate seawater-induced salt stress and promote growth of tomato
Source: Sci Rep. 2022 Apr 4;12:5599. doi: 10.1038/s41598-022-09519-9 (PMC8980105; doi:10.1038/s41598-022-09519-9)
Supplement: Supplementary file 1 — Supplementary Figure 1. [file 41598_2022_9519_MOESM1_ESM.pptx]

## Slide 1
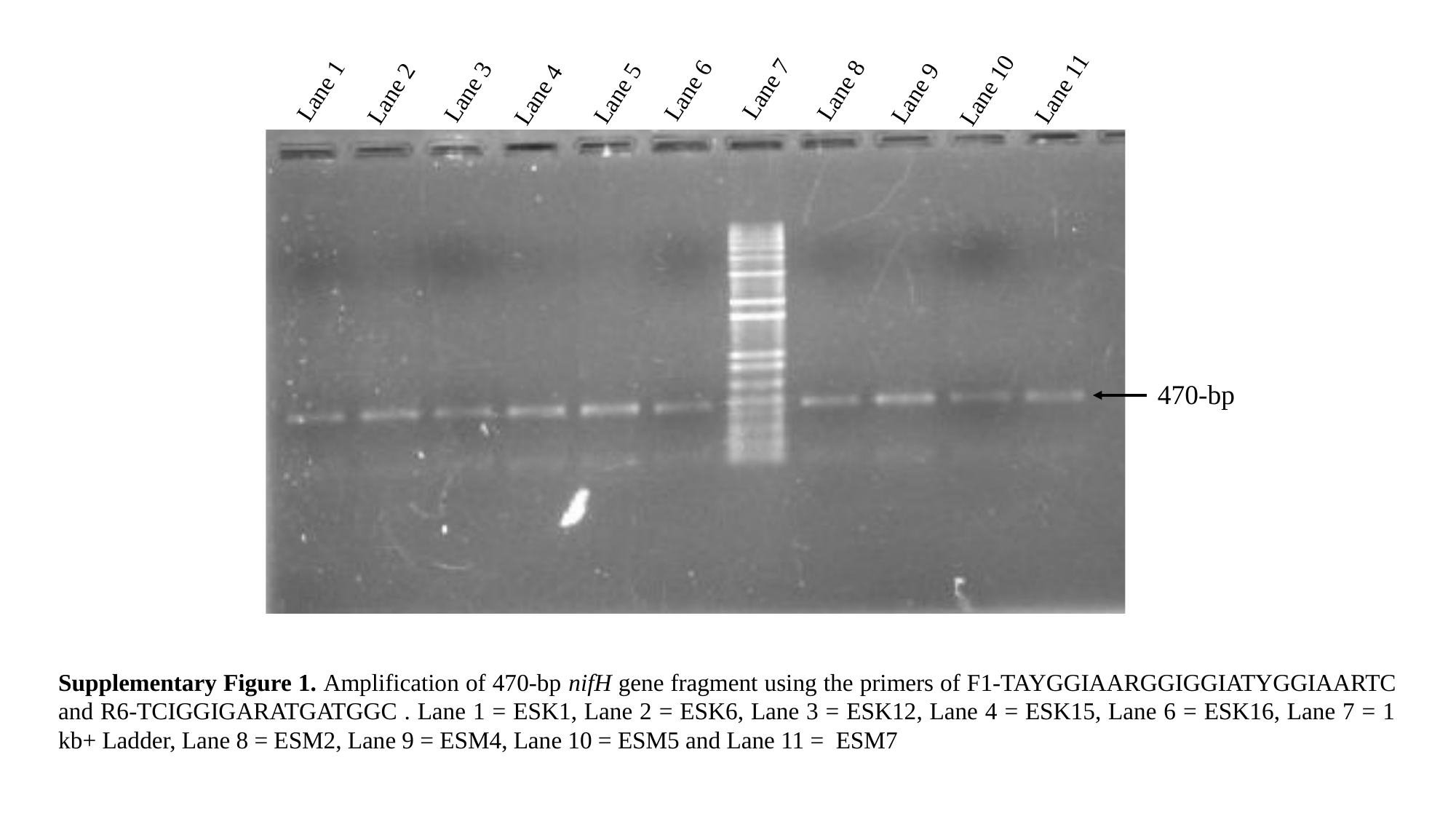

Lane 7
Lane 6
Lane 8
Lane 1
Lane 3
Lane 11
Lane 9
Lane 5
Lane 2
Lane 4
Lane 10
470-bp
Supplementary Figure 1. Amplification of 470-bp nifH gene fragment using the primers of F1-TAYGGIAARGGIGGIATYGGIAARTC and R6-TCIGGIGARATGATGGC . Lane 1 = ESK1, Lane 2 = ESK6, Lane 3 = ESK12, Lane 4 = ESK15, Lane 6 = ESK16, Lane 7 = 1 kb+ Ladder, Lane 8 = ESM2, Lane 9 = ESM4, Lane 10 = ESM5 and Lane 11 = ESM7
