# Supplementary material for: Halotolerant biofilm-producing rhizobacteria mitigate seawater-induced salt stress and promote growth of tomato
Source: Sci Rep. 2022 Apr 4;12:5599. doi: 10.1038/s41598-022-09519-9 (PMC8980105; doi:10.1038/s41598-022-09519-9)
Supplement: Supplementary file 2 — Supplementary Figure 2. [file 41598_2022_9519_MOESM2_ESM.pptx]

## Slide 1
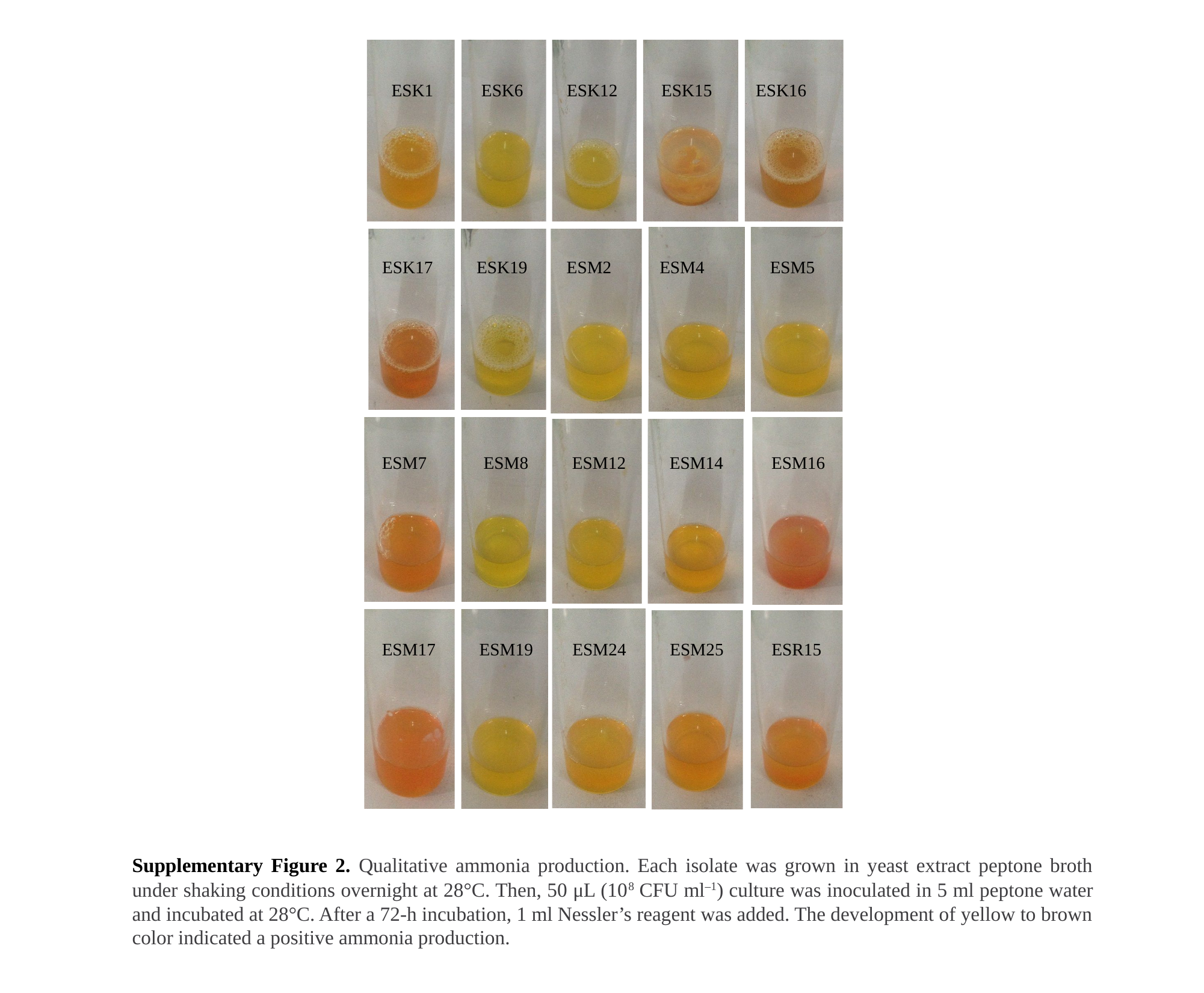

ESK1 ESK6 ESK12 ESK15 ESK16
ESK17 ESK19 ESM2 ESM4 ESM5
ESM7 ESM8 ESM12 ESM14 ESM16
ESM17 ESM19 ESM24 ESM25 ESR15
Supplementary Figure 2. Qualitative ammonia production. Each isolate was grown in yeast extract peptone broth under shaking conditions overnight at 28°C. Then, 50 μL (108 CFU ml–1) culture was inoculated in 5 ml peptone water and incubated at 28°C. After a 72-h incubation, 1 ml Nessler’s reagent was added. The development of yellow to brown color indicated a positive ammonia production.
