# Supplementary material for: Halotolerant biofilm-producing rhizobacteria mitigate seawater-induced salt stress and promote growth of tomato
Source: Sci Rep. 2022 Apr 4;12:5599. doi: 10.1038/s41598-022-09519-9 (PMC8980105; doi:10.1038/s41598-022-09519-9)
Supplement: Supplementary file 3 — Supplementary Figure 3. [file 41598_2022_9519_MOESM3_ESM.pptx]

## Slide 1
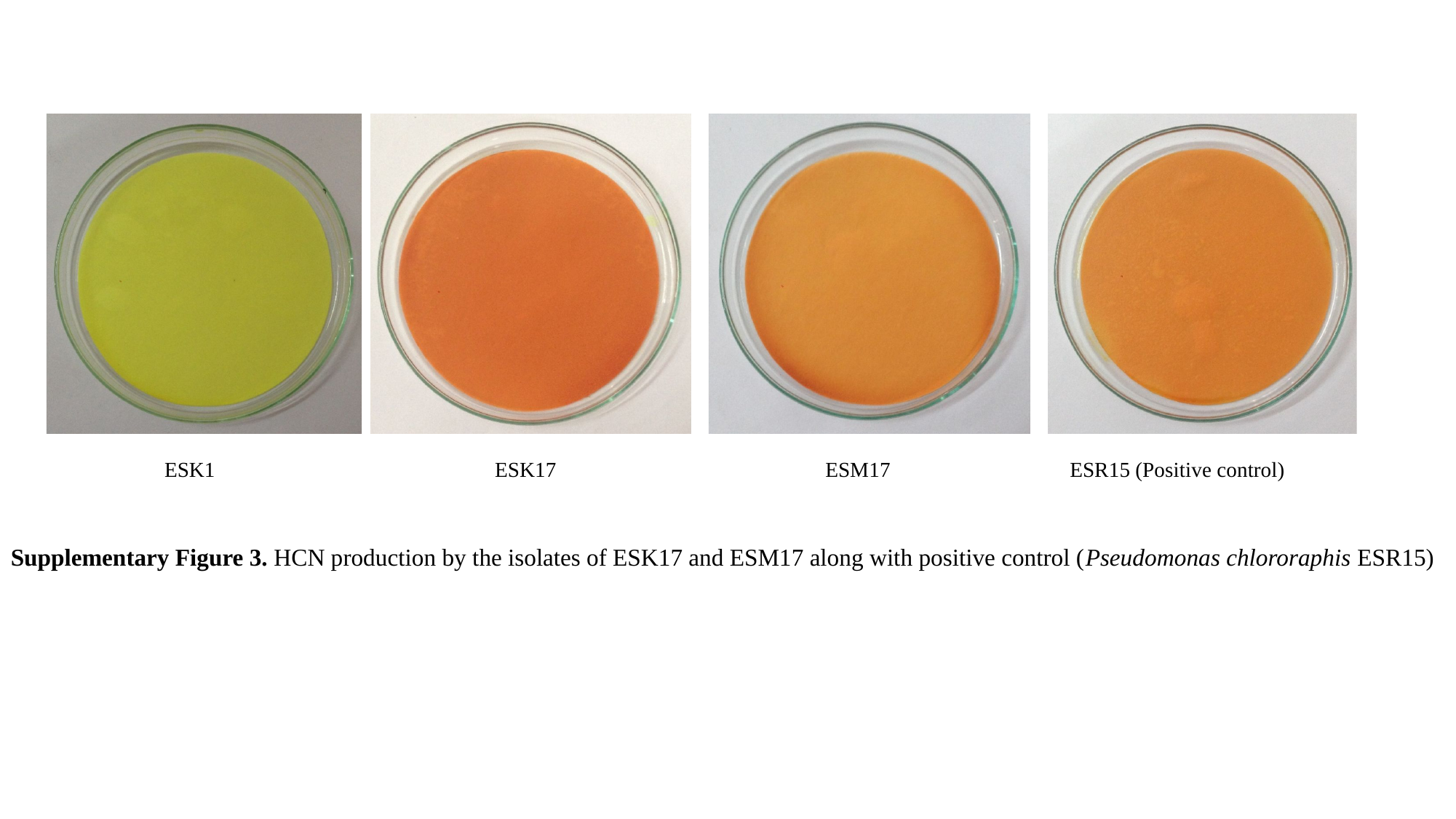

ESK1 ESK17 ESM17 ESR15 (Positive control)
Supplementary Figure 3. HCN production by the isolates of ESK17 and ESM17 along with positive control (Pseudomonas chlororaphis ESR15)
