# Supplementary material for: Halotolerant biofilm-producing rhizobacteria mitigate seawater-induced salt stress and promote growth of tomato
Source: Sci Rep. 2022 Apr 4;12:5599. doi: 10.1038/s41598-022-09519-9 (PMC8980105; doi:10.1038/s41598-022-09519-9)
Supplement: Supplementary file 4 — Supplementary Figure 4. [file 41598_2022_9519_MOESM4_ESM.pptx]

## Slide 1
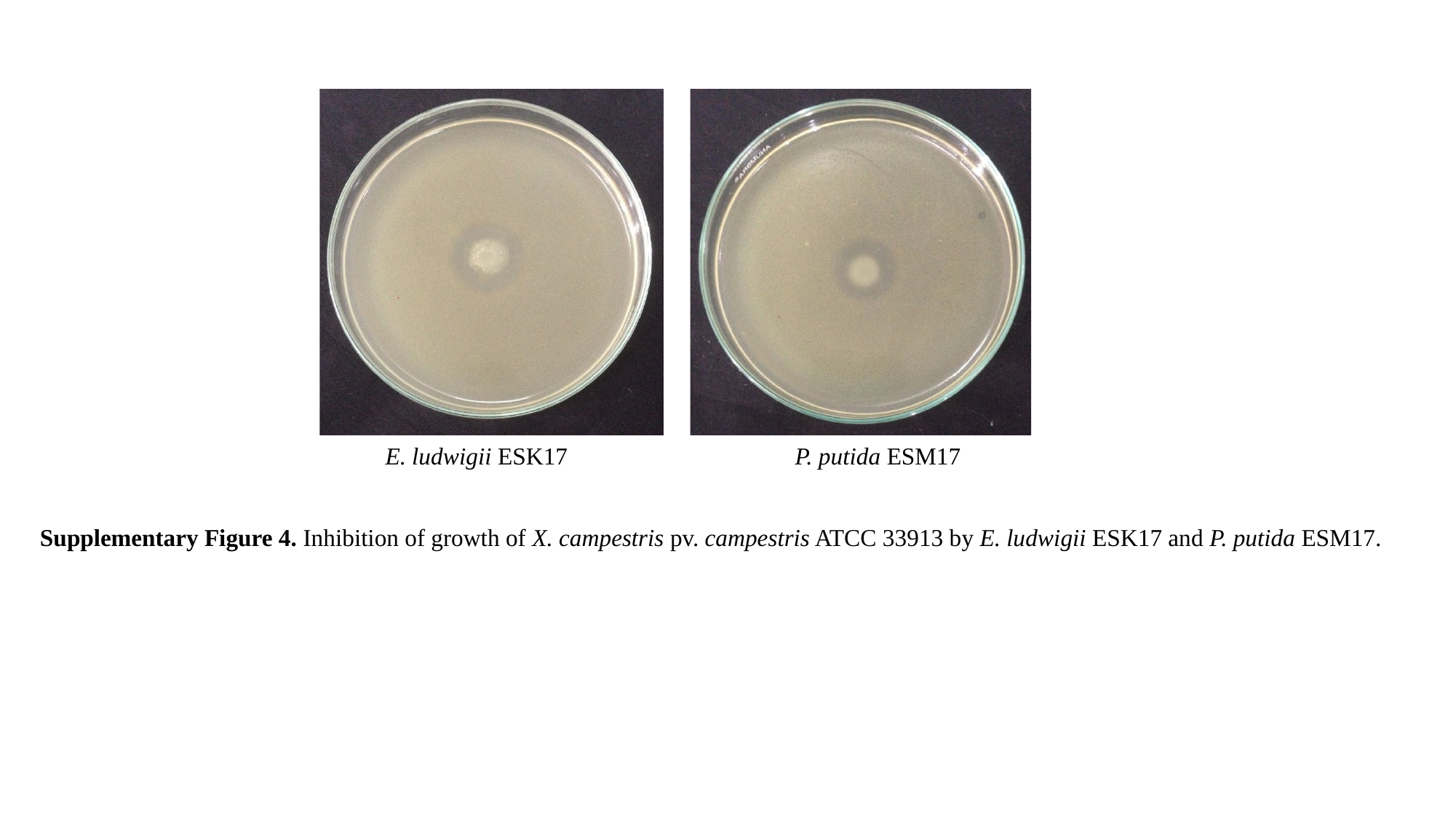

E. ludwigii ESK17
P. putida ESM17
Supplementary Figure 4. Inhibition of growth of X. campestris pv. campestris ATCC 33913 by E. ludwigii ESK17 and P. putida ESM17.
