# Supplementary material for: Halotolerant biofilm-producing rhizobacteria mitigate seawater-induced salt stress and promote growth of tomato
Source: Sci Rep. 2022 Apr 4;12:5599. doi: 10.1038/s41598-022-09519-9 (PMC8980105; doi:10.1038/s41598-022-09519-9)
Supplement: Supplementary file 5 — Supplementary Table 1. [file 41598_2022_9519_MOESM5_ESM.docx]

| Strains | Salinity (% NaCl) | | | pH | | | Temperature (°C) | | |
| --- | --- | --- | --- | --- | --- | --- | --- | --- | --- |
|  | 5 | 10 | 15 | 4.0 | 9.0 | 10 | 37 | 42 | 50 |
| ESK1 | + | + | - | + | + | + | + | + | - |
| ESK6 | + | + | + | - | + | + | + | + | + |
| ESK12 | + | + | - | - | + | + | + | + | - |
| ESK15 | + | + | - | + | + | + | + | + | - |
| ESK16 | + | + | - | + | + | + | + | + | - |
| ESK17 | + | - | - | + | + | + | + | - | - |
| ESK19 | + | + | + | + | + | + | + | + | + |
| ESM2 | + | + | + | - | + | + | + | + | + |
| ESM4 | + | + | + | + | + | + | + | + | + |
| ESM5 | + | + | + | + | + | + | + | + | + |
| ESM7 | + | + | + | - | + | + | + | + | + |
| ESM8 | + | + | + | + | + | + | + | + | + |
| ESM12 | + | + | + | + | + | + | + | + | + |
| ESM14 | + | + | + | + | + | + | + | + | + |
| ESM16 | + | + | + | + | + | + | + | + | + |
| ESM17 | + | + | + | + | + | + | + | + | + |
| ESM19 | + | + | + | + | + | + | + | + | + |
| ESM24 | + | + | + | + | + | + | + | + | + |
| ESM25 | + | + | + | + | + | + | + | + | + |
| ESD3 | + | + | + | - | + | + | + | + | - |

**Supplementary Table 1.** Abiotic stress tolerance of different biofilm-producing bacteria
